# Genomic and transcriptomic insights into molecular basis of sexually dimorphic nuptial spines in *Leptobrachium leishanense*

**DOI:** 10.1038/s41467-019-13531-5

**Published:** 2019-12-05

**Authors:** Jun Li, Haiyan Yu, Wenxia Wang, Chao Fu, Wei Zhang, Fengming Han, Hua Wu

**Affiliations:** 1 Institute of Evolution and Ecology, School of Life Sciences, Central China Normal University, 152 Luoyulu, Hongshan District, Wuhan, 430079 China; 2 Biomarker Technologies Corporation, Beijing, 101300 China

**Keywords:** Molecular ecology, Gene expression, Genome, Conservation genomics

## Abstract

Sexually dimorphic (SD) traits are important in sexual selection and species survival, yet the molecular basis remains elusive, especially in amphibians where SD traits have evolved repeatedly. We focus on the Leishan moustache toad (*Leptobrachium leishanense*), in which males develop nuptial spines on their maxillary skin. Here we report a 3.5 Gb genome assembly with a contig N50 of 1.93 Mb. We find a specific expansion of the intermediate filament gene family including numerous keratin genes. Within these genes, a cluster of duplicated hair keratin genes exhibits male-biased and maxillary skin-specific expression, suggesting a role in developing nuptial spines. We identify a module of coexpressed genes significantly associated with spine formation. In addition, we find several hormones likely to be involved in regulating spine development. This study not only presents a high-quality anuran genome but also provides a reference for studying skin-derived SD traits in amphibians.

## Abstract (web summary)

The basis of sexual dimorphism in non-model species may be elusive, in part due to a lack of genomic and molecular resources. Here, Li et al. report a high-quality anuran genome and reveal candidate genes and pathways associated with shaping sexually dimorphic nuptial spines in a moustache toad.

## Introduction

Males and females of a species often exhibit different strategies to maximize fitness. This difference in optimal strategies can select for phenotypic differences between two sexes^1^. Sexually dimorphic (SD) modifications include male-specific morphology, such as the lion’s mane^2^, the sword-like tail of swordtail fishes^3^, and the greatly enlarged tooth of male narwhals (*Monodon monoceros*)^4^. As most of the genome is shared between sexes with the exception of genes in sex chromosomes, sexual dimorphism is primarily caused by differential, sex-biased gene expression^1^. To achieve sex-biased expression, gene duplication at the genomic level is a potential solution. Redundant paralogs generated by duplication events can allow one or more genes to diverge in expression levels and/or protein structure^5^. A wealth of evidence has proven that duplicated genes often acquire male-biased and tissue-specific expression^6^. Recent studies exploring genome-wide sex-biased expression patterns in different species revealed a broad variation in the percentage of sex-biased genes (ranging from 2% of transcripts in *Littorina saxatilis* to 90% in *Drosophila melanogaster*)^7^. Although these studies provide insights into sex-biased expression patterns, very few of them link sex-biased genes to particular traits. Furthermore, most of conclusions regarding the molecular regulation of SD traits come from studies on model species^8^. The main obstacle linking interesting traits to sex-biased genes outside of model organisms is that we often lack genomic information for nonmodel species, including genomic resource and knowledge of genetic networks governing trait development.

Amphibians are a group of vertebrates with abundant SD traits. In anurans (toads and frogs), >90% of the species exhibit larger body sizes in females than in males^9^. Another type of SD trait in anurans is skin-derived excrescences, such as the nuptial pads on the digits of hands or on the ventral surfaces of forelimbs^10^ and the nuptial spines on the upper jaw^11^. These traits are present mainly in adult males during the breeding period and exhibit a seasonal cycle. A morphological survey on nuptial pads from 26 species in Phyllomedusinae (Hylidae) found that the pads consisted of dark epidermal projections (EPs). The shape and density of EPs and the separation level between adjacent EPs differ among species^12^. Hormone implant experiments revealed that the pads could be induced by androgens in adult males, adult females, and even tadpoles^13^. However, in contrast to the results of morphological examination and hormone manipulation, the genetic basis of shaping nuptial excrescences remains elusive, which can be largely attributed to the lack of genomic information. For instance, a recent study on *Leptobrachium boringii* using nonreference transcriptomic analyses found several processes (such as cytosolic processes and peptidase inhibitor activity) and a list of potential genes (such as insulin-like growth factor genes and sex steroid hormone-related genes) that may be associated with the seasonal development of nuptial spines^14^. However, owing to the absence of a reference genome, the annotation rate of unigenes was very low (30.98%), indicating that most of the unigenes were unannotated^14^. This limitation will hinder our understanding of important biological processes and genes associated with SD traits.

The genomes of amphibians are exceptionally large (up to 120 Gb in salamanders) and feature high levels of repetitive sequences^15^, which makes both sequencing and assembly challenging. To date, six anuran genomes have been sequenced and annotated^16–22^, among which only the genomes of *Xenopus laevis* and *Xenopus tropicalis* have been assembled to chromosome level^17,19^. Among the sequenced anurans, *X. laevis* and *X. tropicalis* belong to the Archaeobatrachia, while the other four species (*Nanorana parkeri*, *Rana catesbeiana*, *Rhinella marina*, and *Oophaga pumilio*) belong to Neobatrachia^23^. However, to the best of our knowledge, no genomic data for the spadefoot toads (Pelobatoidea), a monophyletic clade sister to Neobatrachia relative to the paraphyletic Archaeobatrachia^23^, has been published. Within the Pelobatoidea, Megophryidae that is the most widely diversified family provides an appropriate system for studying skin-derived SD traits in amphibians. For example, in the Leishan moustache toad (*Leptobrachium leishanense*), males have skin excrescences that are similar to skin-derived SD traits in other anurans in terms of tissue structure and developmental cycles. During the breeding period, adult males develop four sharp and conical black spines on the maxillary skin (MS) (two on each side), whereas such a structure is absent in females. During the postbreeding period, adult males lose the conical outer casing^24^. Immature males have only red spots in the same area. It has been proposed that such nuptial spines may be used for male–male combat, stimulation of females, or nest construction/maintenance during the breeding season^25^.

Herein, to build a representative genome of Pelobatoidea and to understand the molecular basis of skin-derived SD traits in amphibians, we focus on the Leishan moustache toad. We first assemble a 3.5 Gb of chromosome-scale genome. Comparative genomic analysis reveals expansion of intermediate filament (IF) gene families (GFs) in *L. leishanense*, which include numerous keratin genes (*krt*s). To identify biological processes and genes associated with the production of nuptial spines, we compare the transcriptomes of multiple tissues, including dorsal skin (DS), MS, brain, and gonad, between males and females at three developmental stages. Stage A is the subadult stage, when males have not developed spines and females have no eggs; stage B is the breeding stage, when males have developed spines and females have eggs; stage C is the postbreeding period, when males’ spines fall off and females have laid eggs. We identify a module of coexpressed genes to be significantly associated with spine formation. In addition, hormones such as androgen, thyroid hormone (TH), prolactin (PRL), and relaxin (RLN) are likely involved in the regulation of spine development. In summary, we obtain a high-quality reference genome and reveal a series of candidate genes underlying the production and regulation of nuptial spines in *L. leishanense*. Similar regulatory patterns can be used to guide future studies on skin-derived SD traits in amphibians.

## Results

### Genome assembly and characterization

**Fig. 1:**
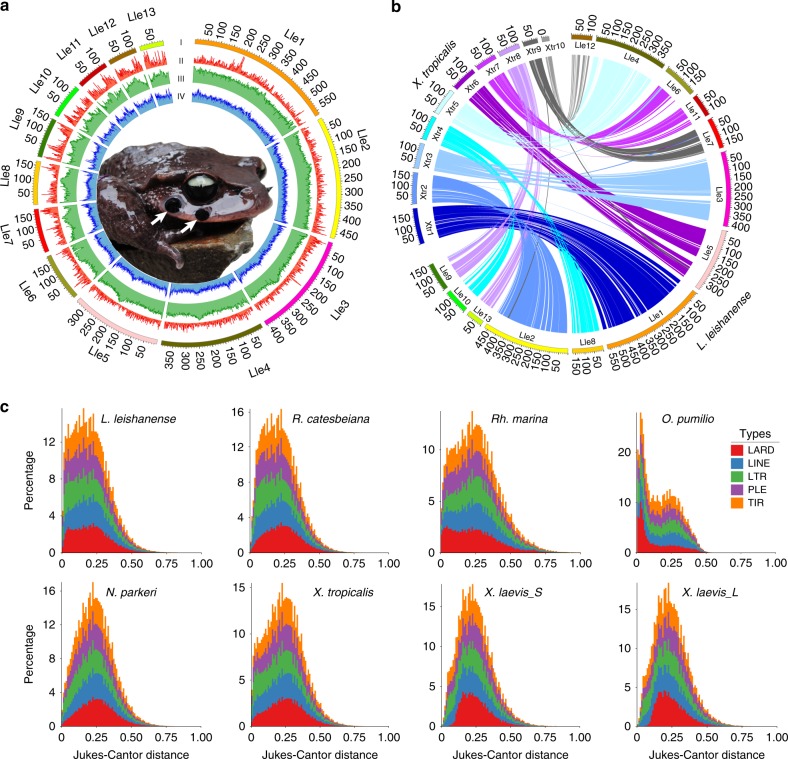
Genomic landscape of *Leptobrachium leishanense* and comparison with other anurans. **a** From outer to inner: (I) sizes of 13 pseudochromosomes; (II) gene density (the percentage of genes in per 200-kb window); (III) repeat sequence distribution; (IV) GC content (%). The photo in the circle shows an adult male with nuptial spines on the upper jaw as marked with two white arrows (two spines on each side). The toad photo was taken by Wei Zhang. **b** Chromosome synteny between *L. leishanense* and *X. tropicalis*. Chromosomes of *X. tropicalis* are marked as “Xtr1–10” and chromosomes of *L. leishanense* are marked as “Lle1–13”. Collinear blocks between two species are linked by lines with the same colors. **c** Divergence distribution of five dominant transposable elements (TEs) in different genomes based on the same analysis procedures (see “Methods”). The *x*-axis is the Jukes–Cantor distance from the consensus in the TE library. The *y*-axis shows percentages of TE occupancy in genomes. LARD large retrotransposon derivatives, PLE Penelope-like elements, TIR terminal inverted repeat.

A male *L. leishanense* toad was selected for genome sequencing and assembly. The genome size was estimated to be 3.56 Gb based on the *k*-mer distribution (Supplementary Fig. 1). A total of 285.81 Gb (~80×) of PacBio long reads were assembled using Canu v1.5^26^ combined with WTDBG v1.1.006 (https://github.com/ruanjue/wtdbg). This assembly was polished based on 174.86 Gb (~50×) of Illumina paired-end reads using Pilon v1.22^27^. Subsequently, we used LACHESIS (http://shendurelab.github.io/LACHESIS/) to assemble contigs on 13 pseudochromosomes^28^ based on 155.16 Gb (~44×) of Hi-C data. A total of 3296 contigs with a length of 3.31 Gb were assembled to chromosome-level scaffolds (Supplementary Fig. 2). We finally generated a 3.54 Gb of *L. leishanense* genome, with a contig N50 of 1.93 Mb and scaffold N50 of 394.69 Mb, providing the first chromosome-anchored genome among Pelobatoidea species (Table 1, Fig. 1a, Supplementary Tables 1 and 2).

**Table 1:** Statistics of assembled genomes among different anurans.

	*Leptobrachium leishanense*	*Nanorana parkeri*	*Rana catesbeiana*	*Rhinella marina*	*Oophaga pumilio*	*Xenopus tropicalis*	*Xenopus laevis*
Assembled genome size (Gb)	3.5	2.1	5.8	2.6	5.5	1.5	2.7
Contig N50 (bp)	1,931,129	32,920	5239	167,489	3942	71,041	19,713
Scaffold N50 (bp)	394,693,044	1,075,367	68,964	167,489	59,503	135,134,832	136,570,856
GC content (%)	43.7	42.5	43.0	43.2	43.5	40.0	40.0
Repetitive sequences (%)	77.1	56.7	66.9	86.6	62.9	51.9	38.3
Protein coding genes	23,420	21,477	22,000	58,302	17,051	27,047	37,385

To evaluate the assembly quality, we mapped the Illumina reads on the reference genome. Approximately 95% of the paired-end reads were mapped properly. Then we aligned 258,442 transcriptomic unigenes from nine *L. leishanense* tissues to the assembly, with >99.6% of unigenes being aligned, indicating excellent coverage of the expressed genes (Supplementary Table 3). Furthermore, we examined the completeness of the conserved core eukaryotic genes (CEGs) and universal single-copy orthologs using CEGMA v2.5^29^ and BUSCO v3^30^ (tetrapoda_odp9 database), respectively. The reference genome of *L. leishanense* includes 241 of the 248 (97.2%) complete CEGs and 3840 of the 3950 (97.2%) complete and partial BUSCO genes, indicating high completeness of the assembled genome. These metrics are higher than those of sequenced anuran genomes as evaluated by the same method (Supplementary Figs. 3 and 4 and Supplementary Tables 4 and 5).

### Genome annotation and chromosome synteny

We annotated the repetitive sequences based on the de novo repeat sequence database of *L. leishanense* combined with Repbase 20.01^31^. We found that 77.1% (2.73 Gb) of the *L. leishanense* genome was repetitive sequences, which is higher than the values for most anurans with sequenced genomes (Table 1). Similar to other species, the most abundant transposable elements (TEs) are terminal inverted repeats (Supplementary Table 6). Notably, two major retrotransposons, long interspersed nuclear element and long terminal repeat (LTR), constitute a higher proportion of the *L. leishanense* genome (24.8% and 17.5%, respectively) than other anurans, suggesting the high accumulation of these TEs in *L. leishanense*. Such accumulation of retrotransposons provides the potential for genome size expansion via duplicative transposition. Moreover, the TE expansion pattern of *L. leishanense* is similar to that of *R. catesbeiana* and *R. marina*, which exhibits one major expansion wave followed by a slight burst that occurred recently (Fig. 1c). After masking repetitive sequences, we annotated a total of 23,420 genes in the reference genome with an average gene length of 30 kb (Supplementary Fig. 5), and 96.4% of these genes were annotated in public databases.

To compare structural characteristics of the genomes between *L. leishanense* and *X. tropicalis*, we analyzed chromosomal synteny based on genome-scale ortholog alignment. We found extensive chromosome synteny between two species, with a vast majority of orthologs located on one chromosome in *X. tropicalis* also being located on a single chromosome in *L. leishanense* (Fig. 1b). In *L. leishanense*, a total of 346 blocks with an average size of 7.68 Mb were collinear with the *X. tropicalis* blocks. The chromosome number in *L. leishanense* is 13, whereas the number in *X. tropicalis* is 10. This difference is reflected in the corresponding chromosomal fissions in *L. leishanense*. Specifically, the blocks on the *X. tropicalis* chromosomes (Xtr4, 7, and 8) are distributed on two separate chromosomes in the *L. leishanense* genome (Lle8+10, 6+11, and 9+13, respectively). In addition, chromosomal rearrangements such as inversion and translocation were detected. In the *L. leishanense* genome, a total of 179 out of 346 blocks were inverted (Supplementary Data 1). Seven regions in *L. leishanense* with sizes of 502 kb–5.93 Mb corresponding to Xtr2, 3, 6, 9, and 10 of *X. tropicalis* were translocated into other non-homologous chromosomes (Fig. 1b, Supplementary Figs. 6 and 7).

### Expanded GFs in *L. leishanense*

**Fig. 2:**
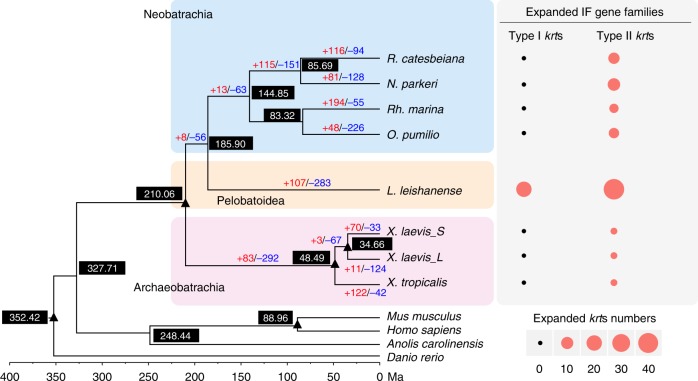
Comparative genomic analyses across anurans. Values in black boxes are divergence times in units of million years ago (Ma, see “Methods”). The number near each branch indicates the number of significantly expanded (red) and contracted (blue) gene families for each clade. Nodes marked with black triangles are used as time calibration nodes. Two intermediate filament (IF) gene families were detected under significant expansion, which include type I and type II keratin genes, respectively. The numbers of expanded keratin genes in different anurans compared to their most recent common ancestor are displayed in circle sizes.

We compared the *L. leishanense* genome with six other anurans (*R. catesbeiana*, *N. parkeri*, *R. marina*, *O. pumilio*, *X. tropicalis*, and *X. laevis*) and four vertebrates (*Mus musculus*, *Homo sapiens*, *Anolis carolinensis*, and *Danio rerio*) to analyze anuran divergence. A phylogenetic tree based on single-copy orthologs revealed the middle position of *L. leishanense* between Neobatrachia and Archaeobatrachia (Fig. 2). The most recent common ancestor (MRCA) among the seven anurans was estimated to occur at 210.06 million years ago (Ma) (176.22–248.83 Ma). *L. leishanense* shared an MRCA with Neobatrachia species at 185.90 Ma (154.10–221.31 Ma, Fig. 2).

It has been proposed that changes in gene copy number would support adaptive evolution^32^. We thus estimated the expanded GFs in *L. leishanense* to explore targets of adaptive evolution. A total of 107 GFs were significantly expanded in *L. leishanense* compared with its MRCA (Fig. 2). Genes from expanded families were mainly enriched in signal response categories, including sensory perception of smell, response to stimulus, and signal transduction (Supplementary Data 2). These categories were also significantly expanded in other anurans such as *N. parkeri*, *R. catesbeiana*, and *R. marina* (Supplementary Data 2). The PI3K-Akt signaling pathway, which plays important roles in responding to extracellular stimuli and regulating multiple cellular functions, such as cell proliferation, apoptosis, and survival^33^, was significantly overrepresented in *L. leishanense* (Fisher’s exact test, Benjamini–Hochberg (BH) corrected *p* < 0.001). In addition, biological processes associated with immune responses, including the immune system process, antigen processing and presentation, and immune response, were significantly and specifically expanded in *L. leishanense* (Supplementary Data 2). Further analyses and empirical data are needed to explore the relationship between habitat adaptation and the expansion of immune response-related genes in *L. leishanense*.

### Expanded keratin genes support nuptial spine formation

We found two GFs encoding IF proteins were significantly expanded in *L. leishanense* (Fig. 2). These GFs include genes encoding keratins that are filament-forming proteins of epithelial cells and are involved in shaping keratinized tissues in vertebrates, such as human hairs and nails^34^. Considering the hardness of nuptial spines, we hypothesize that the expansion of the IF GFs may be associated with the occurrence of nuptial spines in *L. leishanense*. We therefore conducted a comprehensive analysis of *krt*s in *L. leishanense* and compared to other vertebrates.

**Fig. 3:**
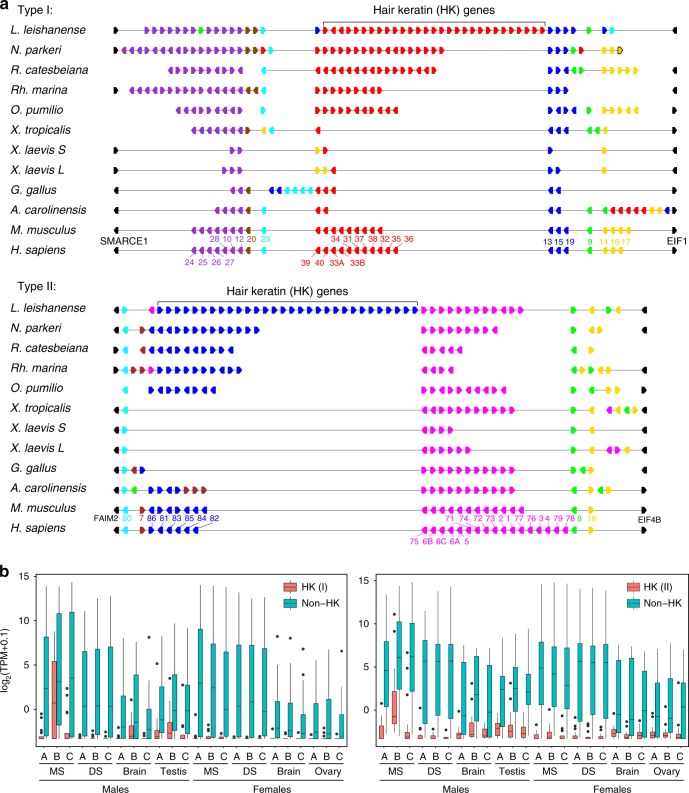
Genomic distribution and expression pattern of the keratin gene family. **a** Genomic organization of type I and type II α-keratin genes in different species. Polygon arrows indicate the direction of keratin genes in the genome. Gene colors are marked based on homologous relationships with human keratin genes. Protein names in human are listed. **b** Box plots of the expression levels for HK genes and non-HK genes in different samples. The expression level was evaluated by log_2_ (TPM + 0.1). For each boxplot of type I HK, *n* = 17 genes’ mean expression values based on three biological replicates; for each boxplot of type I non-HK, *n* = 22 genes; for each boxplot of type II HK, *n* = 17 genes; for each boxplot of type II non-HK, *n* = 15 genes (Supplementary Data 4). The middle line of a box indicates the median (50 percentile) of the expression values for HK (red boxes) and non-HK (blue boxes) genes. The upper and lower terminal lines of a box represent the 25th and 75th percentiles, respectively. Lines extending vertically from the boxes (whiskers) indicate variability outside the upper and lower percentiles.

In tetrapod, an important component of integument rigidity is formed by two classes of α-keratins (termed type I and type II α-keratins)^34^. Genomic analyses have demonstrated that humans possess a total of 54 functional *krt*s (28 type I and 26 type II), separately clustered on two chromosomes^35^. In *L. leishanense*, we identified a total of 101 complete α-*krt*s, including 53 type I and 48 type II genes (Supplementary Data 3), which is much higher than the number observed in other species^34^ (Supplementary Table 7). By comparing the genomic distribution of α-*krt*s, we found that type I and II α-*krt*s were separately clustered on two chromosomes (Lle 12 and Lle 2) in *L. leishanense* and were flanked by the same genes as those in other vertebrates, suggesting conserved arrangement order of *krt*s. There are several clusters of lineage-specific paralogous *krt*s in mammals (human and mouse), *L. leishanense*, and other anurans (Fig. 3a; Supplementary Fig. 8). The most diversified and duplicated *krt*s within *L. leishanense* are homologous to mammalian hair keratins (HKs). Notably, the copy number of HK genes in *L. leishanense* is almost double than in other anurans (Fig. 3a).

To further explore the correlation of duplicated HK genes with nuptial spines in *L. leishanense*, we sequenced the expressed RNAs and compared the expression patterns of HK and non-HK genes in multiple samples (Supplementary Fig. 9). We found that the duplicated HK genes (types I and II) in *L. leishanense* showed male-biased and MS-specific expression during the breeding stage (B), whereas non-HK genes expressed similarly in both the MS and DS in two sexes (Fig. 3b, Supplementary Data 4). These results support that the expansion of the IF GFs, especially the highly duplicated HK genes, is associated with the occurrence of nuptial spines in *L. leishanense*.

### Biological processes and pathways in producing spines

To investigate biological processes associated with nuptial spines, we sequenced transcriptomes from the MS in two sexes and three developmental stages (Table 2). We also sequenced transcriptomes from the DS as controls for skin. For each stage, we performed three groups of independent comparisons: (1) comparison of MS between males and females; (2) comparison of MS against DS in males; and (3) comparison of MS against DS in females. We identified differentially expressed genes (DEGs) for each comparison and conducted Gene Ontology (GO) and Kyoto Encyclopedia of Genes and Genomes (KEGG) enrichments.

**Table 2:** Sampling codes for transcriptomic analyses.

Stages	Dorsal skin	Maxillary skin	Brain	Gonad
A	AM1, AF1	AM2, AF2	AM3, AF3	AM4, AF4
B	BM1, BF1	BM2, BF2	BM3, BF3	BM4, BF4
C	CM1, CF1	CM2, CF2	CM3, CF3	CM4, CF4

*M* male, *F* female

**Fig. 4:**
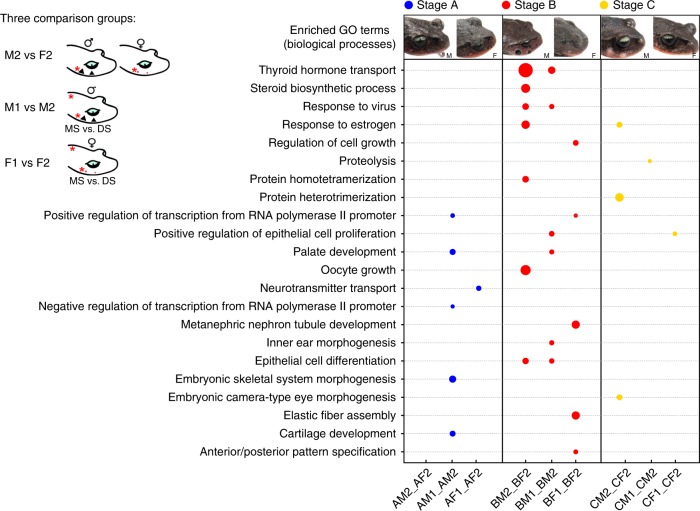
Biological processes associated with producing nuptial spines in *Leptobrachium leishanense*. The legend in the left corner shows three groups of comparisons conducted in each stage: M2 vs F2 indicates the comparison of maxillary skin between males and females (AM2_AF2, BM2_BF2, and CM2_CF2 in the right dot plot panel); M1 vs M2 indicates the comparison of male dorsal skin and maxillary skin (AM1_AM2, BM1_BM2, CM1_CM2); F1 vs F2 indicates the comparison of female dorsal skin and maxillary skin (AF1_AF2, BF1_BF2, CF1_CF2). Red stars on the cartoon toads represent tissues used for comparison in each group. Jun Li made the hand-drawn cartoon toads in Adobe Illustrator CC 2018 with the help of Graphics Tablet (Huion, Model: H420, made in Shenzhen, China). The head figures on the top show the phenotypes of male (M) and female (F) *L. leishanense* toads at three stages. These toad photos were taken by Jun Li. The colored circles in the dot plot represent three stages, and the circle sizes represent enrichment factors with large size indicating a high degree of enrichment.

Stage A is the subadult period, when males and females show similar red spots on the MS. In adult males, these spot regions develop black spines during breeding seasons. Thus DEGs in males’ MS at stage A would be associated with early preparation for spine development. Differential expression analyses revealed that only 19 out of the 15,135 genes were expressed differentially between males’ and females’ MS (|log_2_(fold change)| > 1 and BH corrected *p* < 0.01) and no GO term was enriched (Fig. 4, Supplementary Table 8), which is consistent with the similar phenotypes between two sexes. DEGs between the MS and DS in males were mainly involved in transcriptional regulation processes (including positive and negative regulation of transcription from the RNA polymerase II promoter) and skeletal development-related processes (including embryonic skeletal system morphogenesis and cartilage development). Similar processes were not enriched in the comparison of females (Fig. 4). Stage B is the adult breeding period, when males have developed spines and females retain red spots in the MS. Thus DEGs in males’ MS at stage B would be directly associated with the formation of nuptial spine. We found that 83 out of 14,409 genes were differentially expressed in the MS between two sexes. These DEGs were enriched in hormone activities (such as TH transport and steroid biosynthetic process) and epithelial cell differentiation (Fig. 4). Similar processes were also enriched for DEGs in males’ MS vs DS but were absent in the same comparison for females (Fig. 4). At the postbreeding stage (C) when males’ spines fall off, we found that the DEGs of males’ MS vs DS were significantly enriched in the proteolytic process that contains multiple genes encoding trypsin and cathepsin (Fig. 4). Proteolysis can break peptide bonds and hydrolyze proteins to small polypeptides (https://www.ebi.ac.uk/QuickGO/term/GO:0006508). It was reported that trypsin could degrade human epidermal keratins in vitro^36^. We thus consider that the proteolytic process may be involved in triggering the falling off of the spines, probably by degrading the keratin protein complex.

**Fig. 5:**
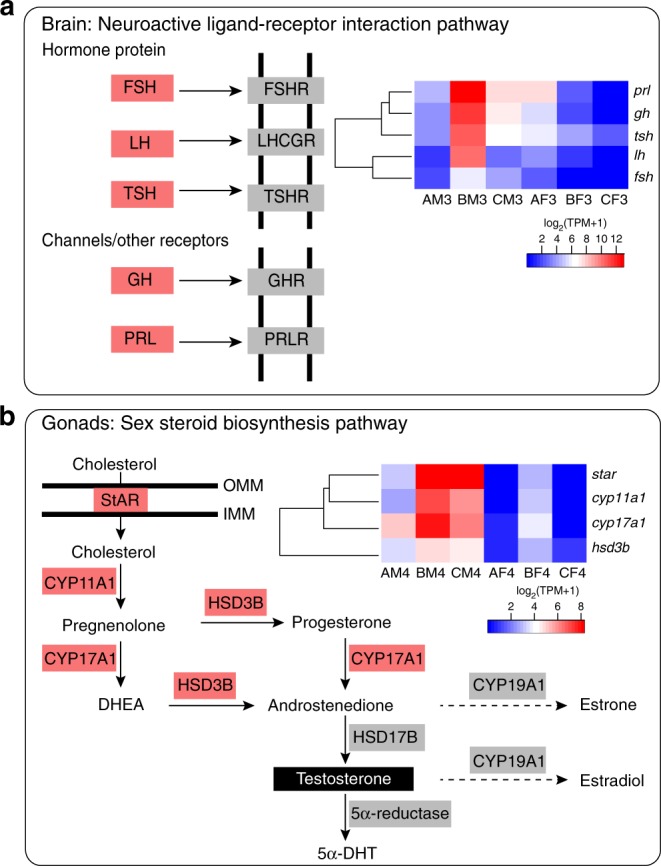
Hormone-related pathways enriched in DEGs of the brain and gonads. **a** An illustration of the neuroactive ligand–receptor interaction pathway. The red boxes are ortholog genes that are highly expressed in male brains at stage B. The heatmap shows the expression levels of five genes across six brain samples. *fsh* follicle-stimulating hormone, *lh* luteinizing hormone, *tsh* thyroid-stimulating hormone, *gh* growth hormone. **b** An illustration of the sex steroid biosynthesis pathway revised from the map00140 in the KEGG database. The red boxes are enzymes that are highly expressed in male testes at stage B. The heatmap shows the expression levels of four genes across six gonad samples. *star* steroidogenic acute regulatory protein, *cyp11a1* cytochrome P450 side-chain cleavage enzyme (P450scc), *cyp17a1* cytochrome P450 17α-hydroxylase/17,20-lyase (P450c17), *hsd3b* 3β-hydroxysteroid dehydrogenase, DHEA dehydroepiandrosterone, 5α-DHT 5α-dihydrotestosterone, OMM outer mitochondrial membrane, IMM inner mitochondrial membrane.

Findings from amphibian endocrine research have revealed that male-biased structures such as enlarged flexor muscles and nuptial thumb pads are androgen dependent^13^. In amphibians, as in other vertebrates, androgens (testosterone and 5α-dihydrotestosterone) are mainly produced by testes and regulated by gonadotropins released from the pituitary gland^37^. Therefore, to explore the hormonal regulation of nuptial spines in *L. leishanense*, we sequenced the transcriptomes from brain and gonads (Table 2). DEGs from brains were significantly enriched in the neuroactive ligand–receptor interaction pathway (BM3 vs BF3 and AM3 vs BM3, Supplementary Fig. 10), which includes numerous signaling molecules, such as hormones and the associated receptors. Within this pathway, five genes encoding hormone subunits were specifically highly expressed in males’ brains at stage B (BM3 in Fig. 5a). Among these genes, the *lh* (luteinizing hormone) and *fsh* (follicle-stimulating hormone) encode peptide hormones that stimulate the synthesis of steroid hormones by traveling via the blood to the gonads^38^. High expression of these genes indicates the activation of the hypothalamus–pituitary–gonad axis in males during the breeding season. Consistently, the steroid hormone biosynthesis pathway was significantly enriched in males’ testes between different stages (AM4 vs BM4 and BM4 vs CM4, Supplementary Figs. 10 and 11). Within this pathway, genes encoding enzymes involved in sex steroid biosynthesis were highly expressed in male testes at stage B (Fig. 5b), suggesting a high level of androgen biosynthesis.

In addition to gonads, the skin is a major nonclassic tissue for steroidogenesis via the expression of functionally active enzymes^39^. In *L. leishanense*, DEGs between the male and female MS were also significantly enriched in the steroid biosynthesis process, which includes genes encoding 3-beta-hydroxysteroid dehydrogenase (*hsd3b*), 17α-hydroxylase (*cyp17a1*), and steroidogenic acute regulatory protein (*star*)^40^. High expression of these enzymes indicates the accumulation of C19 steroids of androgens in males’ MS. Although the level of steroidogenesis in nonclassic tissues is quite modest compared with that in gonads, this process could be very important in locally autocrine or paracrine regulation^39^. However, androgens alone are insufficient to support male SD traits, and other hormones play critical roles as well^13^. Here we found that TH, PRL, and RLN may be involved in the regulatory process. The “thyroid hormone transport” term was highly enriched in DEGs in BM2 vs BF2 and BM1 vs BM2 (Fig. 4). Furthermore, genes encoding PRL and RLN were more highly expressed in the male MS than in the female MS based on both RNA-seq and quantitative PCR (qPCR) data (false discovery rate (FDR)_prl_ < 0.001, log_2_(FC)_prl_ = −8.41; FDR_rln_ < 0.001, log_2_(FC)_rln_ = −10.59, Supplementary Figs. 12 and 13).

### Gene coexpression analyses reveal the genetic networks underlying the spine production

**Fig. 6:**
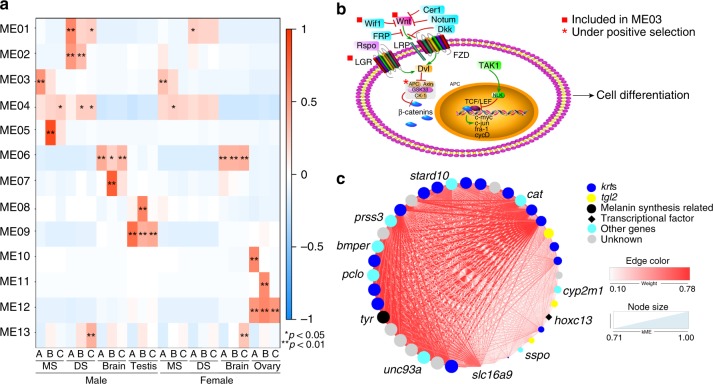
Gene modules correlated with forming nuptial spines in *Leptobrachium leishanense*. **a** Correlation between coexpressed modules and sampling traits. Heatmap colors represent correlation coefficients (*Fisher’s asymptotic *p* < 0.05; **p < 0.01). Rows are different modules, and columns are different sampling traits. **b** An illustration of the canonical Wnt pathway. Genes marked with red squares are included in the ME03 module; the gene marked with a red star is under significant positive selection in *L. leishanense* compared to other anurans. **c** Weighted coexpression network for genes in the ME05 module. Dark-blue circles are keratin genes; yellow circles are genes encoding protein-glutamine gamma-glutamyltransferase 2 (*tgl2*); the black circle is *tyr* gene that is annotated as a melanin synthesis-related gene; the black diamond is a transcriptional factor (*hoxc13*); light-blue circles are other annotated genes involved in diverse metabolic processes; gray circles are unknown genes. Colored lines represent the weight of connectivity between genes, with white to red indicating weights from low (0.10) to high (0.78). The node sizes are associated with the kME values ranging from 0.71 to 1.00. *stard10* StAR-related lipid transfer (START) domain containing 10, *cat* catalase, *tgl2* protein-glutamine gamma-glutamyltransferase 2, *cyp2m1* cytochrome P450 2M1, *hoxc13* homeobox protein Hox-C13, *sspo* SCO-spondin (precursor), *slc16a9* monocarboxylate transporter 9, *unc93a* Protein unc-93 homolog, *tyr* tyrosinase, *pcol* protein piccolo-like, *bmper* BMP-binding endothelial regulator, *prss3* trypsin-3.

To further identify the key genes controlling the production of nuptial spines, we analyzed the gene coexpression networks and identified genetic modules using weighted gene correlation network analysis (WGCNA)^41^. We sought to identify modules (groups of highly correlated genes that may exhibit the same biological activity) that were associated with specific traits. Based on the correlation of expression profiles among genes, we identified 12 modules in total, with the 13th module including genes that were not assigned to any module (Fig. 6a; Supplementary Fig. 14; Supplementary Data 5). Then we characterized the gene expression pattern of each module based on the module eigengene (ME), which represents the first principal component of the scaled module expression profiles. MEs of each module were correlated to external sample traits, with significantly correlated modules suggesting specifically high expression patterns for a particular trait. We found that ME03, representing 170 genes, was significantly correlated with MS at stage A in both males and females (*r*_male_ = 0.53, *p* < 0.001; *r*_female_ = 0.32, *p* = 0.007, Fig. 6a), suggesting that genes in this module are mainly involved in MS specialization prior to spine development. Within this module, positive regulation of the canonical Wnt signaling pathway was significantly enriched (corrected *p* = 0.012, Supplementary Data 6). In addition, the Wnt signaling pathway (ko04310) was enriched from KEGG analysis (*p* = 0.02, corrected *p* = 0.27, Supplementary Data 6). In the canonical Wnt signaling pathway, genes encoding the Wnt protein, two leucine-rich repeat-containing G-protein coupled receptors (LGRs), and Wnt inhibitory factor 1 (Wif1) were included in ME03 (Fig. 6b). The coexpression pattern between the Wnt protein and LGR receptors suggests the activation of Wnt signaling in the MS prior to the formation of nuptial spines. In addition, we analyzed the positively selected genes in *L. leishanense* compared with six other anurans using the branch-site model of CODEML in PAML v4.9^42^. The gene encoding adenomatous polyposis coli, which facilitates β-catenin degradation^43^, had experienced significantly positive selection in *L. leishanense* (*p* = 0.004; Supplementary Data 7). Signaling by the Wnt proteins is one of the fundamental mechanisms that directs cell proliferation during embryogenesis^43^. Moreover, the Wnt signaling pathway has been reported to facilitate the formation of skin appendages such as teeth, hair follicles, and deer antlers by mediating epithelial–mesenchymal interactions and determining cell fate^44,45^. Therefore, we propose that activation of the Wnt signaling pathway is involved in triggering the production of nuptial spines in *L. leishanense*. This result reveals the co-option of the pre-existing Wnt signaling pathway for generating novel traits.

We also found that module ME05 showed a significant correlation with the male MS at stage B (*r* = 0.86, *p* < 0.001, Fig. 6a). This module contains 43 genes, 16 of which are *krt* genes (Fig. 6c). Within this module, the coexpression network reveals that *krt* genes showed strong correlation with other genes, supporting the core roles of *krt* genes in the formation of SD spines. This module also contains three *tgl2* genes (encoding protein-glutamine gamma-glutamyltransferase 2) that are associated with cross-linking in the keratin assembly process^46^. In addition, the *hoxc13* gene showed a distinct correlation with other genes, although the edge weight was not as high as that of the *krt* genes (Fig. 6c). It has been shown that *hoxc13* is involved in the regulation of human HK expression^47^. The gene encoding tyrosinase (*tyr*), which is the essential enzyme in melanin synthesis^48^, was also included in this module, which suggests the role of this gene in the maintenance of the black color of the spines. Thus this module provides the most potential candidate genes that are directly associated with the occurrence of nuptial spines in male *L. leishanense* toads.

## Discussion

In this study, we generated a Pelobatoidea reference genome by combining PacBio long-read sequencing and chromatin interaction scaffolding methods. We detected two recent burst events of TEs in *L. leishanense* genome, which is similar to the genomes of *R. catesbeiana* (5.8 Gb), *R. marina* (2.6 Gb), and *O. pumilio* (5.5 Gb) from Neobatrachia. In contrast, *Xenopus* species (sizes in 1.5–2.7 Gb) from Archaeobatrachia only presented one peak. However, the number of protein-coding genes is similar across these species, indicating that different expansion and accumulation of TEs is one of the major factors contributing to the variation in genome size in amphibians. Diverse genome sizes in amphibians may be driven by adaptive and/or nonadaptive forces. On the one hand, the genome size is strongly associated with developmental rates in Anura. Natural selection will reduce the genome size in environments that foster rapid embryonic and larval development^49^. On the other hand, the evolution of genome size can be affected by neutral factors, such as mutation rates and effective population size^49,50^. For example, the substitution rates of both mitochondrial and nuclear datasets are positively correlated with genome size in Anura^49^. Exploring the relative roles of adaptive and neutral factors in driving changes in genome size would be interesting when an increasing number of amphibian genomes have been sequenced.

Although vertebrate genomes differ greatly in DNA content and chromosome karyotype, extensive synteny exists across species^51^. Here we detected a highly conserved genomic arrangement pattern between *L. leishanense* and *X. tropicalis*, despite the different numbers of chromosomes and the long-term divergence (~210.1 Ma) between two species. Similar genomic synteny analysis has been conducted between *N. parkeri* and *X. tropicalis*^18^. Because of the relatively fragmented assembly of the *N. parkeri* genome (scaffold N50 of 1.1 Mb), most of the collinear blocks that belong to one chromosome in *X. tropicalis* were located on separate scaffolds in *N. parkeri*. For example, the orthologs of chromosome Xtr1 were mapped on scaffolds13 and 18 in *N. parkeri*. Thus fragmented assembly will underestimate the number of collinear blocks identified between two genomes. Despite of the fragmented assembly, whole-genome alignments between *N. parkeri* and *X. tropicalis* still revealed a large amount of synteny^18^. Nevertheless, collinear blocks may vary in orientation or position between species. For example, we detected inversions and translocations in *L. leishanense* compared with the chromosomes of *X. tropicalis*, suggesting the presence of structural variation in amphibian genomes.

In this study, we focused on examining the molecular basis underlying the skin-derived nuptial spines in *L. leishanense* by combining genomic and transcriptomic analyses. A previous study has been conducted on *L. boringii* by analyzing the nonreference transcriptomes of males’ MS, brain, and testes between the breeding (B) and postbreeding (C) stages^14^. Although a total of 1181 DEGs were identified between stages B and C in males’ MS, no nuptial spine-specific GO term was enriched, which may be largely due to the lack of a reference genome. Here we designed a comprehensive sampling strategy by collecting more tissues (added DS as a control) at three developmental stages (added subadult stage A) from two sexes (added the same samples from females). Moreover, the high-quality *L. leishanense* genome provided a solid reference for identifying DEGs and for GO enrichments. Transcriptomic analyses revealed meaningful biological processes associated with shaping nuptial spines, such as epithelial cell differentiation, steroid biosynthesis, and TH transport, which have not been identified previously. In addition, genomic-level examination allowed us to analyze the species-specific duplication of *krt*s and the homologous relationship with HK genes in mammals. Based on the expression pattern of *krt*s, we conclude that duplicated HK genes are closely associated with shaping nuptial spines. The study on *L. boringii* also obtained one *krt* (comp28513_c0) expressed differentially; however, it is difficult to extensively annotate *krt*s based solely on transcriptomic data. Therefore, the reference genome of *L. leishanense* contributes greatly to the exploration of the molecular bases underlying nuptial spine formation.

Notably, the duplication of HK genes also occurs in the other four Neobatrachia anurans (*N. parkeri*, *R. catesbeiana*, *R. marina*, and *O. pumilio*). Most of these terrestrial anurans present skin-derived SD structures, such as tiny nuptial pads on the male chest (*N. parkeri*)^52^ and fingers (*R. catesbeiana* and *R. marina*)^53,54^. The skin of male *O. pumilio* exhibits specialized dorsal color^55^. In contrast, the aquatic *Xenopus* species (*X. laevis* and *X. tropicalis*) only have one type I HK gene and do not have any type II HK gene (Fig. 3a). Such differences in the number and duplication of HK genes between terrestrial and aquatic anurans suggest that HK genes may experience species-specific divergence and support the formation of diverse structures in the adaptation to terrestrial habitats in amphibians. Therefore, extensive studies on keratin GFs across amphibians are required.

However, there remain several unanswered questions. First, sex chromosomes that differ between males and females are important to understand the molecular mechanisms underlying sexual dimorphism. Insights obtained from model species, particularly fruit flies, proved that the central mechanism underlying the generation of SD traits is the integration of sex-determination system and the genetic networks governing trait development^8^. Sex chromosomes are usually associated with sex determination. Thus identifying sex chromosomes is crucial for screening genes that control the development of sexual dimorphism. Unfortunately, based on available data, we cannot identify sex chromosomes due to the lack of morphologically distinguishable sex chromosomes in *L. leishanense*^28^. In fact, the sex chromosome system varies considerably in amphibians, and the heterogametic sex (either XX/XY or ZZ/ZW) can vary even between populations of a single species^56^. Pool sequencing based on confidently sexed males and females from multiple populations and assembly to the reference genome may be helpful for identifying sex-specific molecular markers and localizing sex chromosomes. However, the success of pool sequencing depends on the size of detectable differences between sex chromosomes. Very small genetic differences may be difficult to accurately screen.

In addition to the differences in gene content on sex chromosomes, the secretion of sex steroids (androgens and estrogens) differs between males and females in vertebrates, which is another factor affecting sexual dimorphism. Here we found two pathways involved in the hypothalamus–pituitary–gonad regulation axis were significantly enriched in the brain and testes, suggesting a high level of androgen biosynthesis in male testes at stage B. In addition, three genes involved in testosterone synthesis were present at significantly high levels in the male MS during spine development, suggesting local accumulation of androgens in MS. These findings reveal the essential role of androgens in the formation of nuptial spines. However, it is difficult to directly identify the regulatory processes of androgens in triggering the expression of nuptial spine-related genes.

We also observed the involvement of other hormones, such as PRL and RLN, supported by sex-related DEGs in the MS. PRL, which was named for its role in the promotion of lactation in mammals, has been found to serve multiple roles in reproduction^57^. Interestingly, PRL was reported to induce the formation of nuptial pads in combination with TH in male red-spotted newts^58^. Thus we propose that PRL plays an essential role in regulating the production of nuptial spine in *L. leishanense*. RLN is another small peptide hormone that was broadly implicated in regulating the female reproductive process and improving spermatogenesis in vertebrates^59,60^. However, its role in the regulation of SD trait development has rarely been reported^14^. We found that the gene encoding RLN is highly and specifically expressed in the male MS, suggesting the crucial role in nuptial spine production in *L. leishanense*. Further studies on hormone regulation are needed to examine the roles of different hormones in regulating nuptial spine development in *L. leishanense*.

## Methods

### Samples used for different sequencing methods

The Leishan moustache toad samples used in this study were collected from the Leishan county, Guizhou Province, China. For genomic sequencing, we collected muscle from one adult male. Muscle and other eight tissues from the same individual (including DS, MS, brain, testis, liver, heart, kidney, and spleen) were collected for transcriptomic sequencing. For the Hi-C library preparation, we collected 4 ml of heart blood from four adult males to achieve enough sample volume. For comparative transcriptomic sequencing, RNA was extracted from four types of tissues (MS, DS, brain, and gonads) in two sexes at three developmental stages (Supplementary Fig. 9). Each sample includes three biological replicates. For the qPCR validation, we used MS and DS from adult males and adult females during the breeding season. For detailed information on sampling strategies, please see Supplementary Table 9. All experiments involving animals in this study were approved by the Animal Ethics Committee of the School of Life Sciences, Central China Normal University (CCNU-IACUC-2019–008). We have complied with all relevant ethical regulations for animal testing and research.

### Illumina sequencing and genome survey

Muscle tissue from an adult male was flash frozen in liquid nitrogen. Genomic DNA was extracted using the DNeasy Blood & Tissue Kit (Qiagen, Valencia, CA, USA). Eight paired-end libraries and 12 mate-pair libraries were prepared following Illumina protocols. Library sequencing was performed on the Illumina HiSeq 4000 system (Illumina, San Diego, CA, USA). After removal of sequencing adapters, contaminant reads (mitochondrial, bacterial, and viral sequences), and low-quality reads, we finally obtained 572.38 Gb (~163× coverage) of clean reads (Supplementary Table 10). We estimated the genome size by *k*-mer distribution (*k* = 21, Supplementary Fig. 1).

### Sequencing of PacBio long reads and Hi-C libraries

Genomic DNA was sheared by a g-TUBE device (Covaris, Woburn, MA, USA) setting with 20 kb. The sheared DNA was purified and concentrated with AmpureXP beads (Agencourt, Beverly, MA, USA) and further used for Single Molecular Real Time (SMRTbell) library preparation according to the PacBio 20-kb template preparation protocol. The isolated SMRTbell fractions were purified and used for primer and polymerase (P6) binding according to the manufacturer’s binding calculator (Pacific Biosciences, Menlo Park, CA, USA). Single-molecule sequencing was conducted on a PacBio RS-II platform with C4 chemistry. After adapter removal, we totally obtained 285.81 Gb of subreads (80.3× coverage), with an average length of 10.03 kb.

Hi-C libraries were created from the whole blood cells (WBCs) from adult males. According to the protocol, nuclear DNA from WBCs was cross-linked and enzymatically digested with Hind III, leaving pairs of distally located but physically interacting DNA molecules attached to each other. The sticky ends of the digested fragments were biotinylated and ligated to each other to form chimeric circles. Biotinylated circles, which are chimeras of physically associated DNA molecules from the original cross-linking, were enriched, sheared, and sequenced with the Illumina HiSeq 2500 platform. A total of 517.9 million clean Hi-C reads pairs (155.16 Gb, 44× coverage) were obtained.

### Genome assembling processes

We first used Illumina reads (163×) and PacBio long reads (10×) to obtain the initial assembly (this version is referred to as Illumina-and-PacBio Assembly, IPA, Supplementary Table 1). The paired-end reads were assembled to contigs using Platanus^61^. The resulting contigs and PacBio reads were assembled to hybrid contigs with DBG2LOC^62^. Then we used SSPACE^63^ to assemble contigs to scaffolds based on the mate-pair reads, which resulted in the IPA version of the reference genome with a contig N50 of 408.18 kb and scaffold N50 of 754.32 kb (Supplementary Table 1). Considering the poor quality of the IPA version and the high proportion of repeat sequences estimated from the genome survey, we chose to use pure PacBio reads to assemble the genome of *L. leishanense*.

We used the 285.81 Gb of PacBio reads to assemble the second version of genome. Prior to assembly, we used the error correction module in Canu v1.5 to correct long subreads. Sequencing errors were corrected with a corrected error rate of 0.025. The corrected subreads were used for genome assembly using WTDBG v1.1.006. The draft genome was polished using Pilon v1.22 with 174.86 Gb of paired-end reads, which produced a *L. leishanense* reference genome with a contig N50 of 2.29 Mb (this version is referred to as PacBio-and-Illumina Polishing, version PIP, Supplementary Table 1).

Then we used the Hi-C data to correct the PIP version and assembled contigs to chromosome-level scaffolds. We used the Hi-C interaction signals to correct assembling errors. Contigs within the assembled genome were broken into fragments with a length of 50 kb. These fragments were used for correcting errors by reassembling based on Hi-C interaction signals. We regarded those positions that cannot be restored to the original positions on the assembled genome as candidate error regions. Within these regions, the positions with low Hi-C coverage were identified as error points. Based on the Hi-C correction, the contig N50 of the PIP version decreased slightly from 2.29 to 1.93 Mb. Then we assembled 3296 (out of 8601) contigs to 13 pseudochromosomes. We finally generated a 3.54 Gb of the *L. leishanense* reference genome, with a contig N50 of 1.93 Mb and scaffold N50 of 394.69 Mb (this version is referred to as PacBio-Illumina polishing and Hi-C assembly, version PIH, Supplementary Table 1), which was used for subsequent analyses.

### Evaluation of genome assembly

To evaluate the quality of the *L. leishanense* reference genome, we aligned the Illumina reads onto the assembly. We also aligned 258,442 unigenes (≥100 bp) from 9 tissues on the assembly to assess the completeness of gene regions. In addition, we identified conserved eukaryotic core genes and single-copy orthologs in tetrapods based on the CEGMA v2.5 and BUSCO v3 databases, respectively. Based on the standard evaluation procedures, we searched 208 (out of 248, 83.9%) CEGs and 3142 (out of 3950, 79.5%) BUSCO genes in the assembly. To further check whether the missing genes exist in the assembled genome, we aligned the predicted genes in our genome against the hmmer profiles of missing genes in the CEGMA and BUSCO databases, respectively. For the CEGMA evaluation, we defined the genes with >70% of coverage as the complete CEGs based on the standard procedure. We finally found 33 complete CEGs presented in our assembly, which were missed in the standard evaluation procedure. To further explore why these CEGs cannot be detected based on the standard procedure, we examined the features of missing genes and found that these genes usually have longer introns (Supplementary Fig. 3). For the BUSCO evaluation, we also searched the missing genes based on the hmm model. We finally identified 421 previously missed BUSCO genes in *L. leishanense* genome, which resulted in 97.22% of complete and fragmented BUSCOs. Similar with the missing CEGs, the missing genes that omitted by original BUSCO evaluation procedure are featured by long introns compared with the complete BUSCOs. Similar phenomenon also exists in other anuran genomes (Supplementary Fig. 4).

### Genome annotation and chromosome synteny analysis

We constructed the de novo repetitive sequence database of *L. leishanense* and combined with the Repbase database 20.01 to create the final repeat library (for detailed software list, please see Supplementary Table 6). Repeat sequences in the *L. leishanense* genome were identified and classified using RepeatMasker 4.0.6^64^. The LTR family classification criterion was defined by the 5’LTR sequences of the same family that shared at least 80% identity over at least 80% of their lengths. To compare repeat sequences across different anurans, we applied the same methods for six other genomes (*N. parkeri*, *R. catesbeiana*, *X. tropicalis*, *X. laevis*, *R. marina*, and *O. pumilio*).

We integrated three approaches, namely, de novo prediction, homology search, and transcript-based assembly, to annotate protein-coding genes in a repeat-masked genome (for detailed software list, please see Supplementary Fig. 5). Consensus gene models were generated by integrating the de novo prediction and protein and transcript alignments using EVidenceModeler v1.1.1^65^. To assign gene functions, the predicted gene sequences were searched against seven databases: NR, GO, KEGG, KOG, Pfam, SwissProt, and TrEMBL.

We analyzed the chromosome synteny between *L. leishanense* and *X. tropicalis* via all-to-all BLASTP searches of protein sequences (with an *E*-value cut-off of 1e−5). Collinear blocks containing at least 10 genes (-s 10) and a maximum of 25 gaps (genes) between two proximal orthologs within a block (-m 25) were identified using MCScanX^66^. The serial numbers of the chromosomes were manually adjusted to reflect the descending order of chromosome length (Lle1 is the longest chromosome of *L. leishanense*, and Lle13 is the shortest chromosome).

### Comparative genomic analyses

Orthologous groups among 11 species (including *D. rerio*, *A. carolinensis*, *H. sapiens*, *M. musculus*, *N. parkeri*, *X. tropicalis*, *X. laevis*, *O. pumilio*, *R. marina*, *R. catesbeiana*, and *L. leishanense*) were constructed using OrthoMCL v2.0.9^67^ based on an all-to-all BLASTP strategy (with an *E*-value of 1e−5). We extracted 881 single-copy genes from the 11 species and aligned proteins for each gene. All the alignments were combined to one supergene to construct a phylogenetic tree using RAxML v7.2.8 with 1000 rapid bootstraps followed by a search of the best-scoring maximum likelihood (ML) tree in one single run. Divergence time was estimated using the MCMCTree program in PAML v4.9 under the relaxed clock model. Several calibrated time points were used to date the divergence time in the unit of Ma (Supplementary Table 11).

A GF was defined as a group of similar genes that descended from a single gene in the last common ancestor. Expansion and contraction of GFs were determined using CAFÉ v3.1^68^ based on changes in GF size. The cluster size of each branch was compared with the cluster size of the ancestral node. *p* value was calculated using the Viterbi method under the hidden Markov model, with *p* < 0.05 defining significant expansion or contraction. Genes belonging to expanded GFs were subjected to GO and KEGG enrichment. *p* values were calculated with two-sided Fisher’s exact test and corrected by the BH procedure.

To identify positively selected genes (PSGs) in *L. leishanense*, we first extracted the orthologous genes of *L. leishanense* and six other anurans via reciprocal best alignment. The protein sequences were aligned and removed sites with gaps. The alignments were used for PSG identification. We used the branch-site model of CODEML in PAML v4.9 by setting the *L. leishanense* as the foreground branch and the six other anurans as background branches. We allowed *ω* values (the ratio of the rate of nonsynonymous substitutions to the rate of synonymous substitutions) to vary among sites in the foreground clade following three categories (0 < *ω*_0_ < 1; *ω*_1_ = 1; *ω*_2_ > 1). The likelihood of M2a was compared with that of the null model M1a (0 <*ω*_0_ <1 and *ω*_1_ = 1) by performing a likelihood ratio test (defined as twice the log likelihood difference between the M2a and M1a) and calculating the corresponding *p* values. Sites showing a signature of positive selection were identified by calculating the posterior probability that a site belongs to a category with *ω* > 1 using the Bayes Empirical Bayes approach. Genes with *p* < 0.05 and containing at least one codon with a posterior probability >0.95 were defined as PSGs.

### Keratin GF analyses

The structures of keratin genes in *L. leishanense* were determined using the software GeMoMa v1.4.2^69^ based on the keratin gene models available from the reference organisms (*H. sapiens*, *M. musculus*, *A. carolinensis*, *Gallus gallus*, *X. laevis*, *X. tropicalis*, and *N. parkeri*). The predictions were handled using a GeMoMa annotation filter with default parameters except for the evidence percentage filter (*e* = 0.1), and then the predicted *krt* genes were manually checked to obtain a single high confidence transcript prediction per locus. Keratin genes in *R. catesbeiana*, *O. pumilio*, and *R. marina* genomes were also searched using GeMoMa v1.4.2. Then the keratin gene sequences from the 11 species were aligned and used to construct an ML tree in RAxML v7.2.8.

### Transcriptomic analyses

Samples from the DS, MS, brain, and gonads were collected at three developmental stages from males and females. Each sample included three replicates; thus we collected a total of 72 samples (Supplementary Fig. 9). Total RNA was isolated using the TRIzol reagent (Invitrogen, Carlsbad, CA, USA) followed by treatment with RNase-free DNase I (Promega, Madison, WI, USA) according to the manufacturers’ protocols. RNA quality was checked using an Agilent 2100 Bioanalyzer. Illumina RNA-seq libraries were prepared for 72 samples and sequenced on a HiSeq 2500 system with a PE150 strategy following the manufacturer’s instructions.

The expression level of predicted transcripts in each RNA-seq library was calculated as the transcripts per million (TPM) using the following formula: TPM = (CDS read count × mean read length × 10^6^)/(CDS length × total transcript count). To identify sex-related DEGs, we compared TPM values from various tissues between males and females. Taking MS (tissue #2) as an example, we separately compared AM2 vs AF2, BM2 vs BF2, and CM2 vs CF2. Similar comparisons were conducted for the brain and gonads. In addition, to examine genes specific to the MS, we used DS (tissue #1) as a background and compared MS vs DS in males and females separately (in males: AM1 vs AM2; BM1 vs BM2; CM1 vs CM2; in females: AF1 vs AF2; BF1 vs BF2; CF1 vs CF2). For each pairwise comparison, DEGs were identified by |log_2_(fold change)| > 1 and BH corrected *p* < 0.01 in the DESeq package^70^. The potential functions of the DEGs were examined by GO and KEGG enrichment.

### qPCR validation

To validate the accuracy of DEGs identified from transcriptomic data, we selected five genes and tested the relative expression levels of these genes between the males DS and MS at stage B (BM1 vs BM2) and the expression levels between the males’ and females’ MS at stage B (BM2 vs BF2) using qPCR. The reactions were performed in a CFX96 Touch RealTime PCR Detection System (Bio-Rad, Richmond, CA, USA) using TransStart Tip Green qPCR SuperMix (TransGen, Beijing, China) in a 15-µl volume with 1.5 µl of cDNA. The expression levels of the tested genes were normalized to the level of *gapdh* (glyceraldehyde-3-phosphate dehydrogenase). Two-sided Student’s *t* test was used to evaluate the significance of differential expression between samples. Primers are listed in Supplementary Table 12. Three biological replicates were examined for each sample.

### Gene coexpression analysis

To cluster genes with similar expression patterns across samples, we conducted coexpression analysis based on 72 samples using WGCNA v1.63. We constructed an unsupervised network for transcriptome data using the function blockwiseModules with default parameters. First, a matrix of Pearson correlations between genes was generated based on TPM values across samples. Then an adjacency matrix representing the connection strength among genes was constructed by raising the correlation matrix to a soft threshold power to achieve a scale-free topology fit index of 0.80. Next, the adjacency matrix was used to calculate the topological overlap matrix (TOM). Genes with similar coexpression patterns across samples were grouped using hierarchical clustering of dissimilarity among the topological overlap measures (1 − TOM). Coexpressed modules were determined using a dynamic tree cutting algorithm setting with a minimum module size of 30 and a cut height of 0.998. For each module, GO and KEGG enrichments were conducted to understand the enriched functions. An eigengene value (the first principal component of the scaled module expression profiles) was calculated to characterize the overall expression trend for each module. The intramodular connectivity was measured as kME values that represent the Pearson correlation between the expression level of that gene and the ME. Then the Pearson correlations between ME values and sampling trait values were calculated to measure the strength and direction of association between modules and traits. Fisher’s asymptotic *p* values were calculated for given correlations using the corPvalueFisher module. Significant module–trait associations were considered when *p* < 0.05.

### Reporting summary

Further information on research design is available in the Nature Research Reporting Summary linked to this article.

## Supplementary information

Supplementary Information

Description of Additional Supplementary Files

Supplementary Data 1

Supplementary Data 2

Supplementary Data 3

Supplementary Data 4

Supplementary Data 5

Supplementary Data 6

Supplementary Data 7

Reporting Summary

## Data Availability

All the data have been deposited in the NCBI database under the BioProject PRJNA505224. Specifically, the assembled version IPA of the Leishan moustache toad genome has been deposited in NCBI Genbank (accession: RXON00000000). The HDF5 raw data for PacBio sequencing have been deposited in NCBI SRA database (accession numbers: SRR8897348–SRR8897543; SRR9670029–SRR9670067). The RNA-seq reads for 72 samples have been deposited in NCBI SRA database (accession numbers: SRR8736149–SRR8736220). The Hi-C library reads (including eight libraries) have been deposited in the SRA (accession numbers: SRR8784800–SRR8784807). The Illumina paired-end reads have been deposited in the SRA (accession numbers: SRR8788204–SRR8788209; SRR10019514–SRR10019515). The Illumina mate-pair reads have been deposited in the SRA (accession numbers: SRR10019502–SRR10019513). All these raw data can be downloaded under study SRP188598 [https://trace.ncbi.nlm.nih.gov/Traces/sra/?study=SRP188598]. The annotation files can be found in Figshare (https://figshare.com/; 10.6084/m9.figshare.8019986). Other miscellaneous information are available from the corresponding authors upon request. Published genome data used in the analyses can be found under the following accession codes: *X. tropicalis* (GCF_000004195.3); *X. laevis* (GCF_001663975.1); *N. parkeri* (GCF_000935625.1); *R. catesbeiana* (GCA_002284835.2); *O. pumilio* ([https://academic.oup.com/mbe/article/35/12/2913/5106668#supplementary-data]); *R. marina* (5524/100483); *D. rerio* (GCF_000002035.6); *A. carolinensis* (AnoCar2.0 [ftp://ftp.ensembl.org/pub/release-90/fasta/anolis_carolinensis/dna/]); *M. musculus* (GRCm38 [ftp://ftp.ensembl.org/pub/release-90/fasta/mus_musculus/dna/]); *H. sapiens* (GRCh38 [ftp://ftp.ensembl.org/pub/release-90/fasta/homo_sapiens/dna/]).
