# Supplementary material for: Genomic and transcriptomic insights into molecular basis of sexually dimorphic nuptial spines in Leptobrachium leishanense
Source: Nat Commun. 2019 Dec 5;10:5551. doi: 10.1038/s41467-019-13531-5 (PMC6895153; doi:10.1038/s41467-019-13531-5)
Supplement: Supplementary file 1 — Supplementary Information [file 41467_2019_13531_MOESM1_ESM.pdf]

Supplementary information

Genomic and transcriptomic insights into molecular basis of sexually dimorphic nuptial spines in *Leptobrachium leishanense*

By Li *et al*.

Supplementary Figures

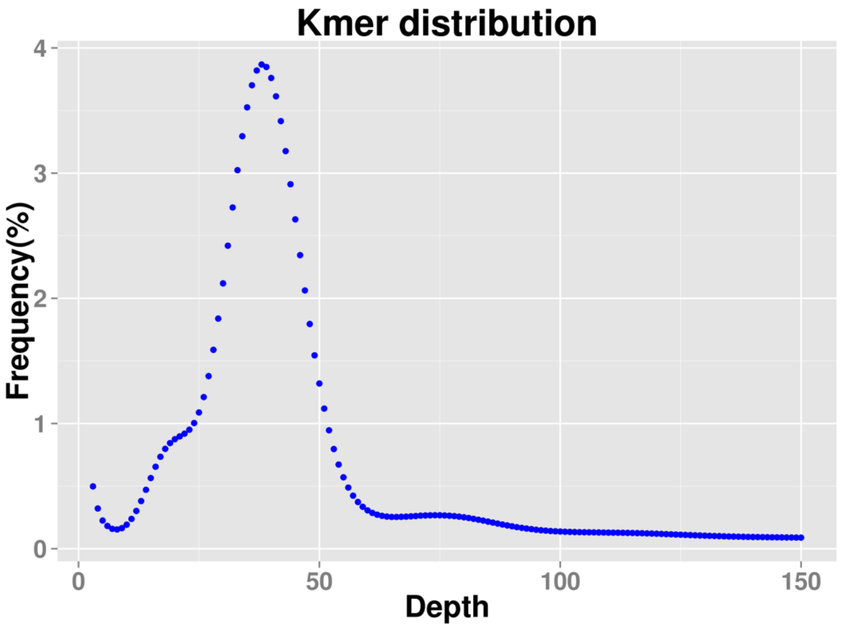

Supplementary Figure 1. Genome size estimation based on *k*-mer distribution. We estimated the genome size by *k*-mer distribution (*k*=21). Based on the Illumina paired-end reads, we obtained a total of 133,264,754,668 *k*-mers, with an average coverage depth of 37.44. Thus, the genome size was estimated to be 3.56 Gb according to the formula: genome size=*k*-mer total number/peak depth.

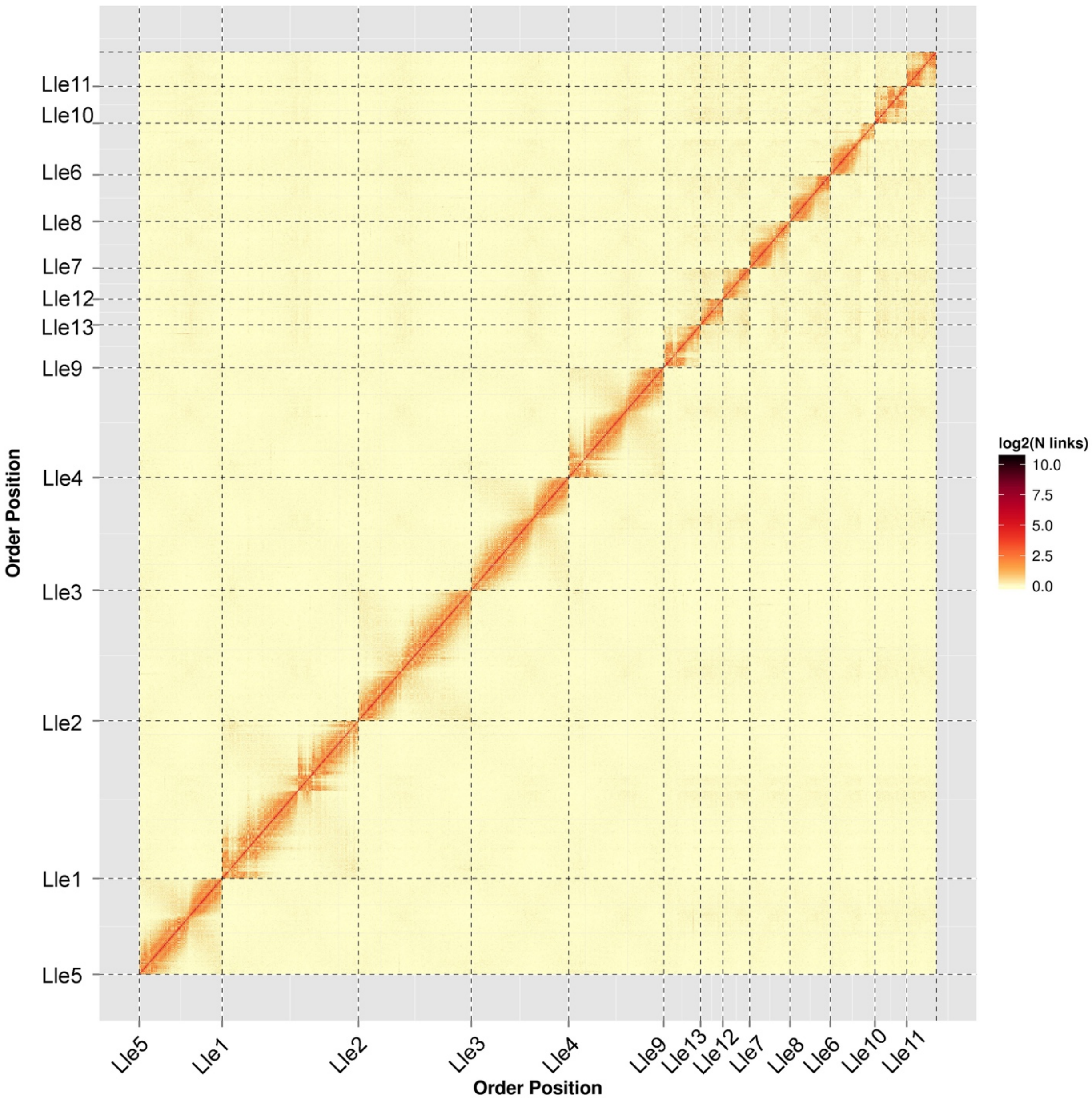

Supplementary Figure 2. The heatmap of Hi-C interaction signals. The Hi-C assembling quality was evaluated by the interaction signaling heatmap, with obviously higher intensity of interaction signals within a chromosome indicating good assembling quality.

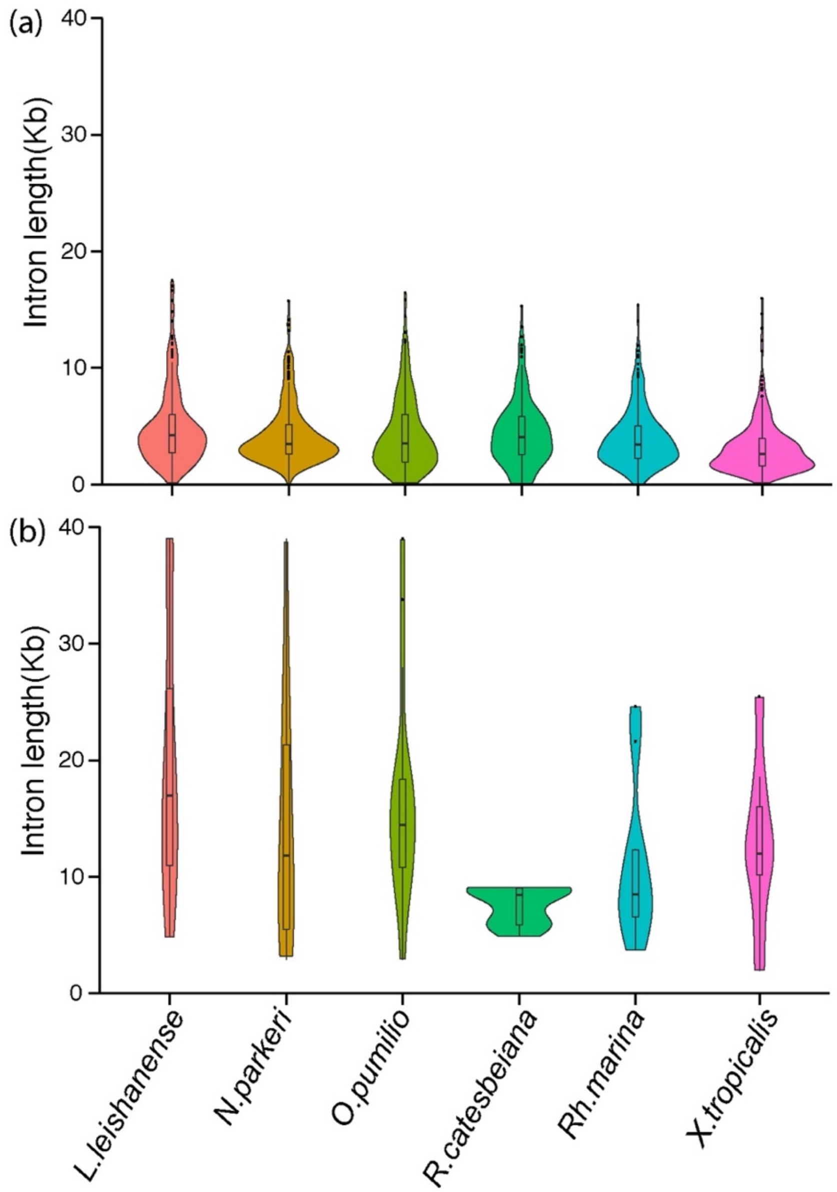

Supplementary Figure 3. Intron length distribution of core eukaryotic genes (CEGs) in different anurans. (a) complete CEGs detected by standard evaluation process. (b) newly detected missing CEGs. Based on the standard evaluation procedures, we found 208 (out of 248, 83.9%) CEGs in the assembly. To further check if the missing genes exist in the assembled genomes, we aligned all the predicted genes in different anuran genomes against the hmmer profiles of missing genes in the CEGMA database[1]. We finally found 33 complete CEGs presented in our assembly, which were missed in the standard evaluation procedure. These missing genes usually have longer introns. Violin plots are the length density of the longest intron of CEGs. Box plots within the violin plots show the median and variation of the longest intron length of missing CEGs in different species. The middle line of a box means the median (50 percentile) of the longest intron length, and the upper and lower terminal lines of a box represent the 25th and 75th percentiles, respectively. Lines extending vertically from the boxes (whiskers) indicate variability outside the upper and lower percentiles.

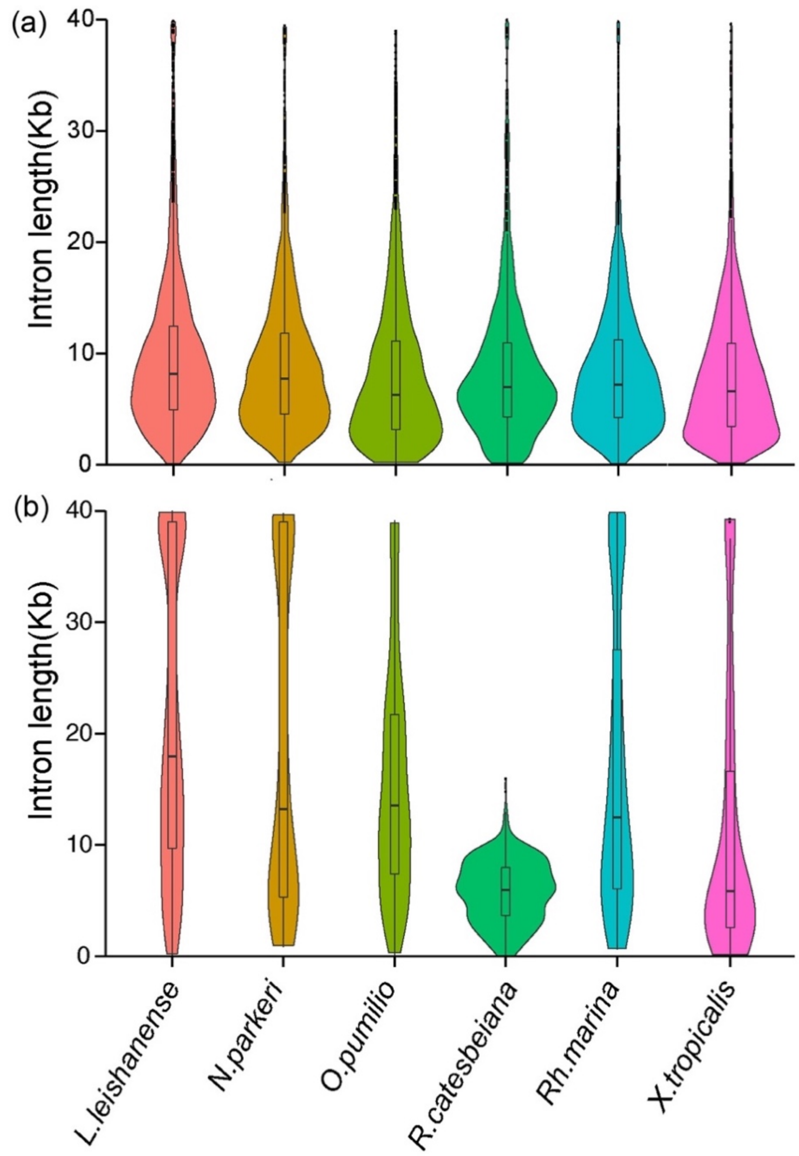

Supplementary Figure 4. Intron length distribution of BUSCO genes in different anurans. (a) complete BUSCO genes detected by standard evaluation process. (b) newly detected missing BUSCO genes. For the BUSCO evaluation, we also searched the missing genes across annotated gene sets based on the hmm model. Then, we calculated sigma values for the genes with the score in hmmer output higher or equal to the scores_cutoff in tetrapod_odb9 database[2]. If -2 <= sigma <= 2, the genes would be identified as complete BUSCOs. After searching the missing BUSCOs across the annotated gene sets, we identified 421 previously missed BUSCO genes in *L. leishanense* genome. These missing genes are featured by long introns compared with the complete BUSCOs. Violin plots are the length density of the longest intron of BUSCO genes. Box plots within the violin plots show the median and variation of the longest intron length of missing BUSCO genes in different species. The middle line of a box means the median (50 percentile) of the longest intron length, and the upper and lower terminal lines of a box represent the 25th and 75th percentiles, respectively. Lines extending vertically from the boxes (whiskers) indicate variability outside the upper and lower percentiles.

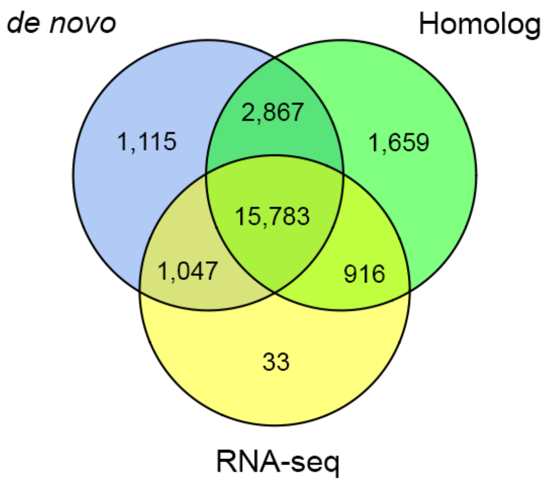

Supplementary Figure 5. Estimated gene numbers based on three methods. We used Genscan v1.0[3], Augustus v2.5.5[4], GlimmerHMM v3.0.1[5], GeneID v1.3[6] and SNAP[7] with default parameters to *de novo* predict genes. All of these software programs were trained using the *X. tropicalis* gene models before gene prediction. Homologous peptides from *D. rerio*, *X. tropicalis*, *X. laevis* and *N. parkeri* were aligned to our genome to identify homologous genes with GeMoMa v1.4.2[8]. The transcripts from nine tissues of *L. leishanense* were aligned to the repeat-masked genome using BLAT[9] and modeled the gene structures using the program PASA v2.0.4[10]. Consensus gene models were generated by integrating the *de novo* predictions and protein and transcripts alignments using EVidenceModeler v1.1.1[11].

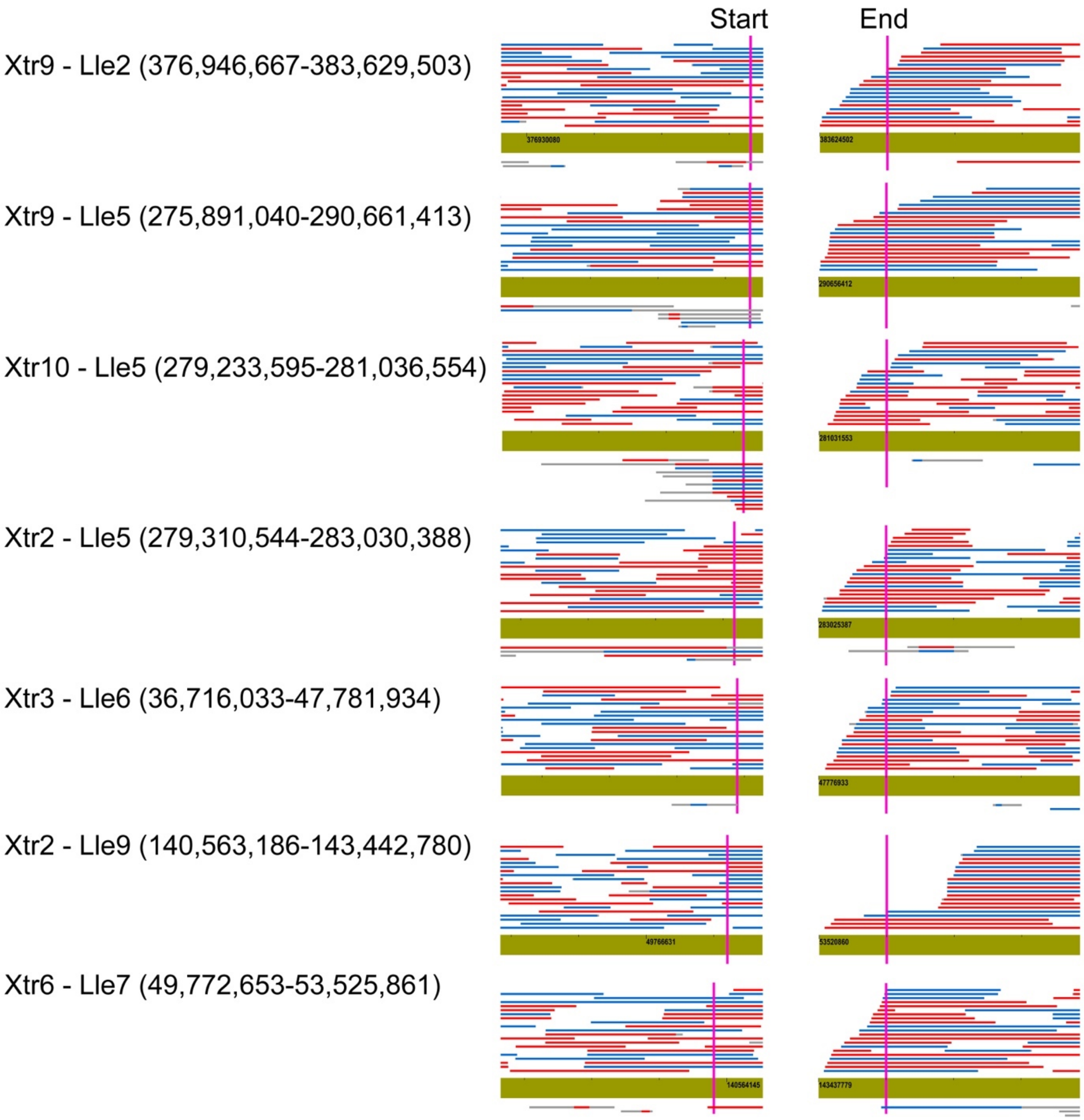

Supplementary Figure 6. Validation of chromosome translocations between *L. leishanense* and *X. tropicalis*. The vertical lines are the locations of start and end sites of colinear blocks. The red lines are PacBio reads with the same direction of the assembly, and the blue lines are reads with the opposite direction of the assembly. To validate the chromosome translocations, we defined the breakpoint locations according to the coordinates of orthologs. Then, the breakpoints were validated by checking the coverage of PacBio reads. The seven inter-chromosomal translocations were validated by the high coverage of PacBio long reads.

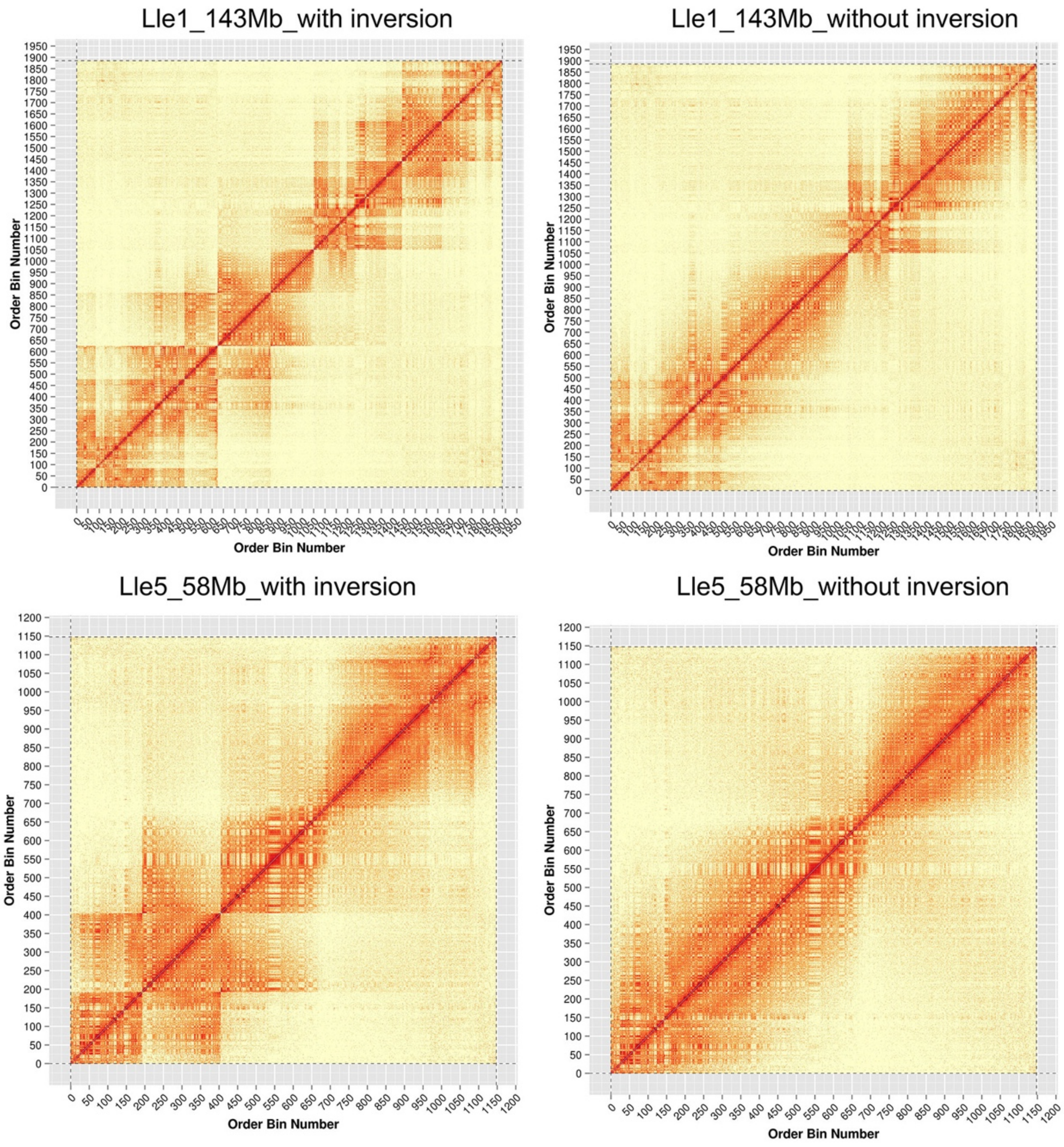

Supplementary Figure 7. Validation of chromosome inversions in *L. leishanense*. We checked breakpoints of chromosome inversions based on the coverage of Hi-C interaction signals. Because of the resolution limitation of Hi-C signals, small size of variations such as the translocations in *L. leishanense* genome (in average size of 6.4 Mb) can not be clearly validated. Thus, we only confirmed larger inversions. We separately generated the interaction signal heatmaps for chromosomes with inversion and without inversion (by manually changing the direction to the same orientation as that in *X. tropicalis*). If the heatmap of chromosomes with inversion presented a better distribution along the diagonal compared with the heatmap of non-inversion chromosomes, the inversion was validated.

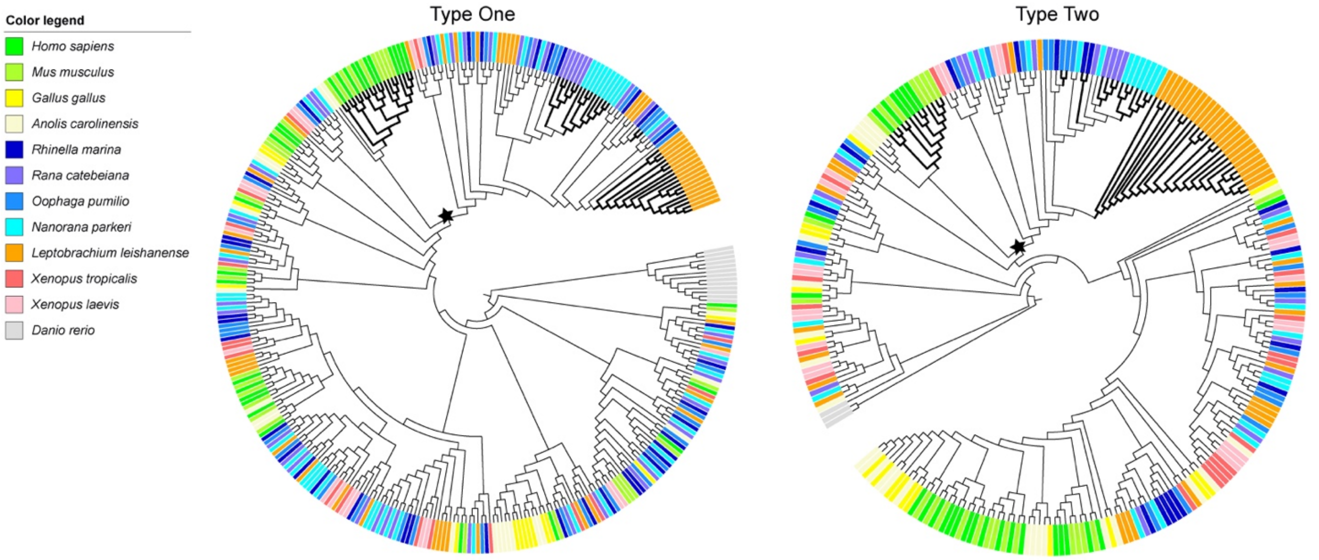

Supplementary Figure 8. Phylogenetic relationships of type I and type II α-keratin genes across vertebrates. Each branch in the tree is one keratin gene. Different colors represent different species. The clades marked with black stars are hair keratin genes based on the homologous relationship with human hair keratins. Bold clades show duplicated hair keratin genes in mammals and anuran species.

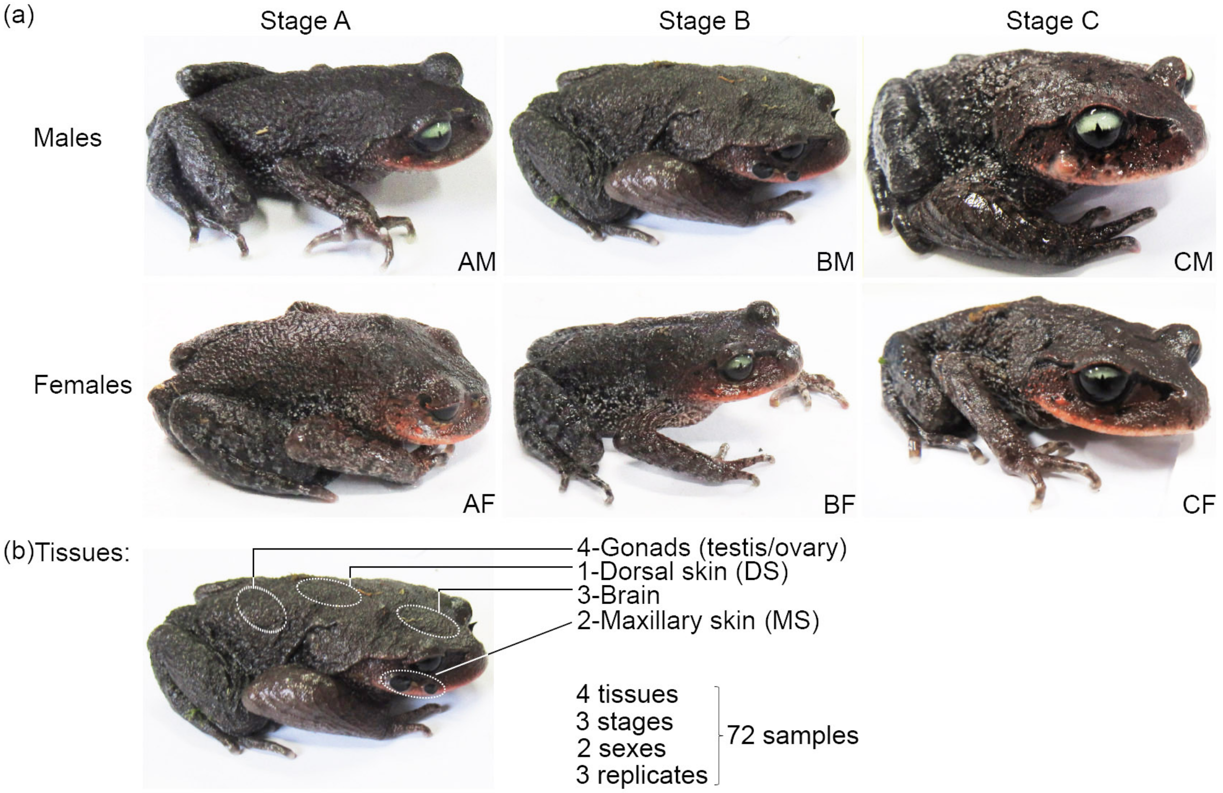

Supplementary Figure 9. Illustration of transcriptome sampling strategy. (a) toad images representing three different development stages (A, B and C) in males (M) and females (F). AM means male samples from stage A, BM means male samples from stage B, CM means male samples from stage C, AF means female samples from stage A, BF means female samples from stage B, CF means female samples from stage C. (b) illustration of tissues collected for each sample. For comparative transcriptomic sequencing, we sampled four types of tissues (1-dorsal skin, DS; 2-maxillary skin, MS; 3-brain and 4-gonads) in two sexes at three developmental stages. Each sample includes three biological replicates. So we totally obtained 72 samples for transcriptomic sequencing. All these toad photos were taken by Jun Li before dissecting.

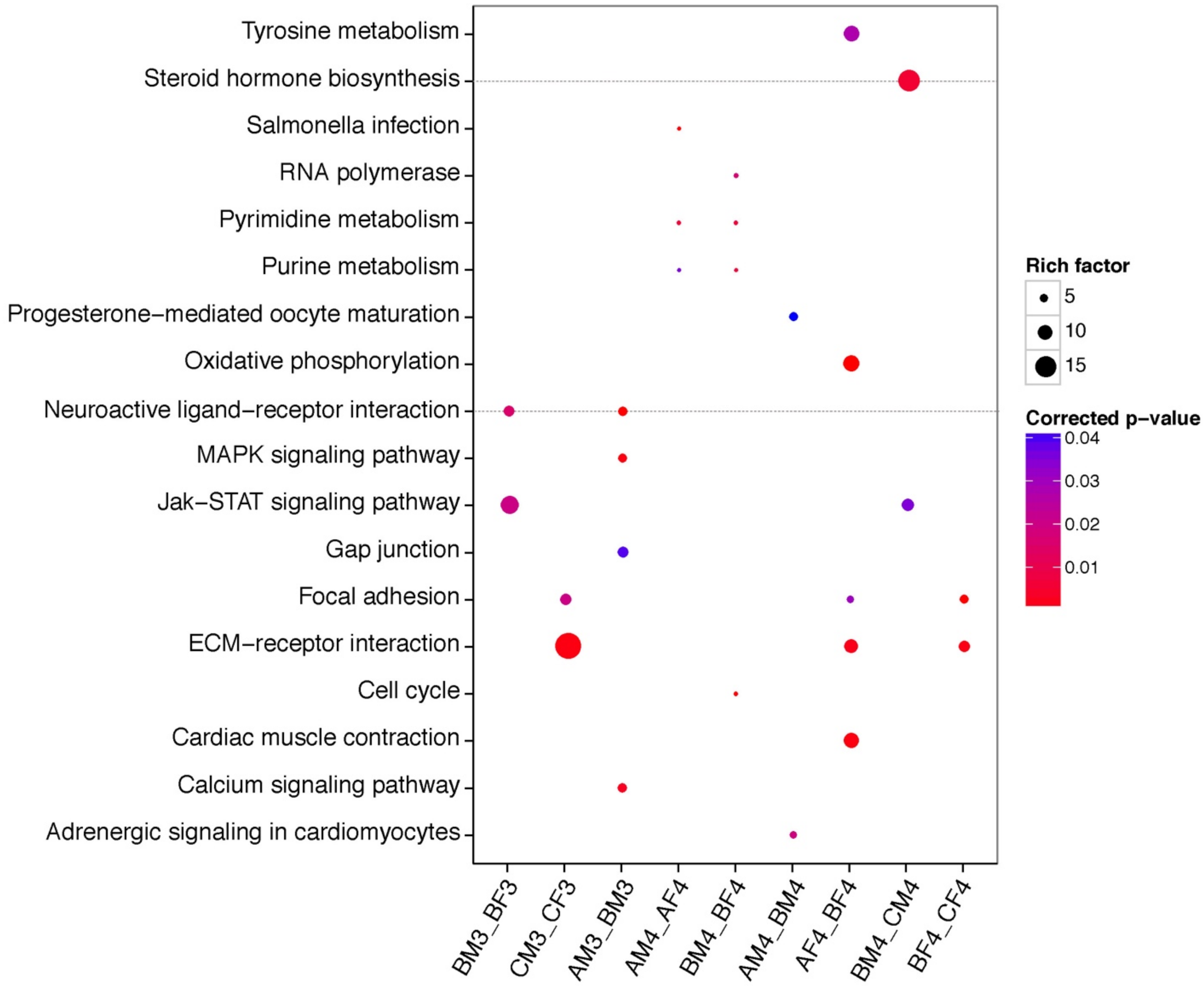

Supplementary Figure 10. Significantly enriched pathways from DEGs in brain and gonad. A, B, C are three developmental stages; M and F stand for males and females, respectively; and tissue 3 is brain, tissue 4 is gonad.

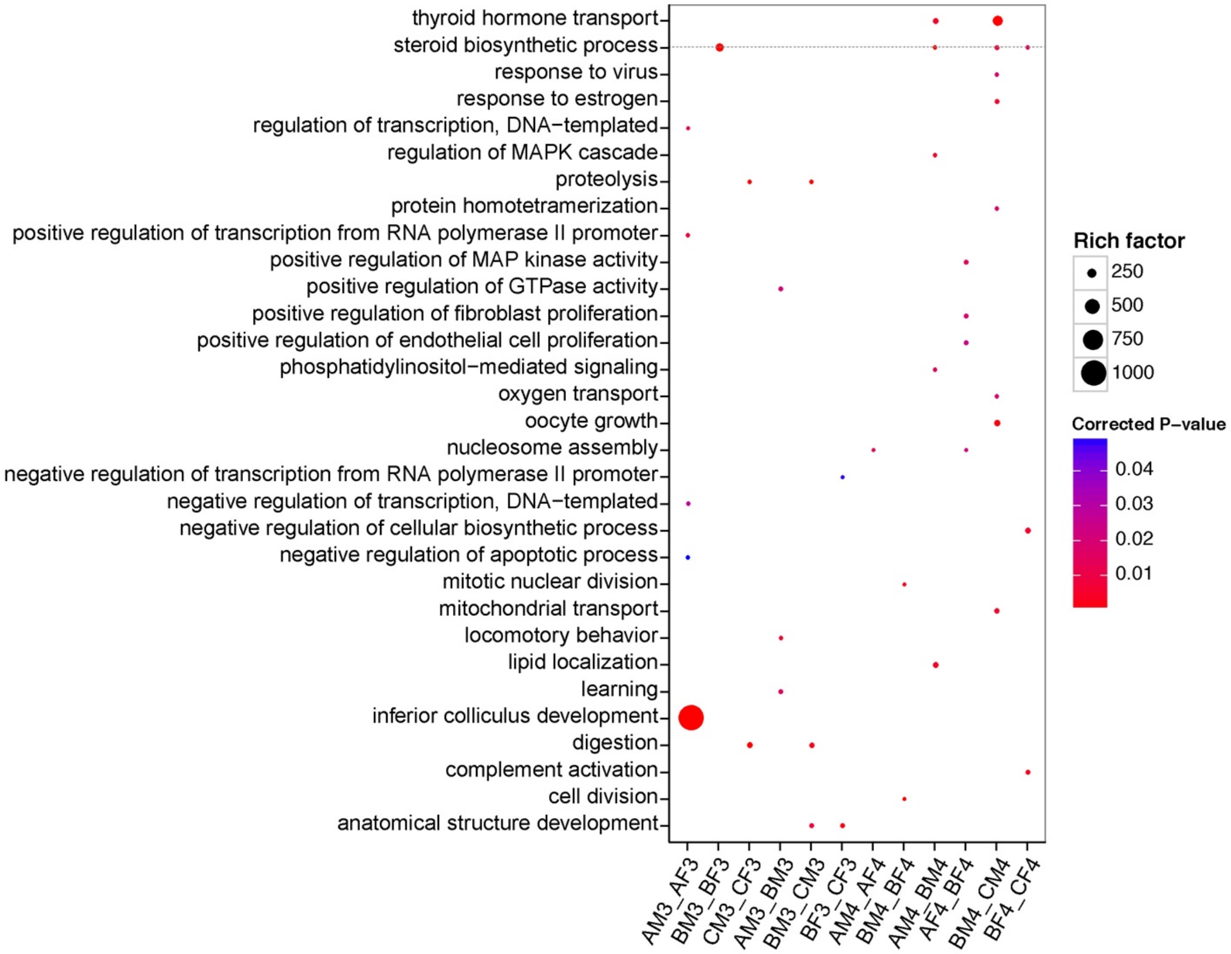

Supplementary Figure 11. Significantly biological processes from DEGs in brain and gonad. A, B, C are three developmental stages; M and F stand for males and females, respectively; and tissue 3 is brain, tissue 4 is gonad. The steroid biosynthesis process is significantly enriched in multiple comparisons (BM3 vs BF3, AM4 vs BM4, BM4 vs CM4 and BF4 vs CF4).

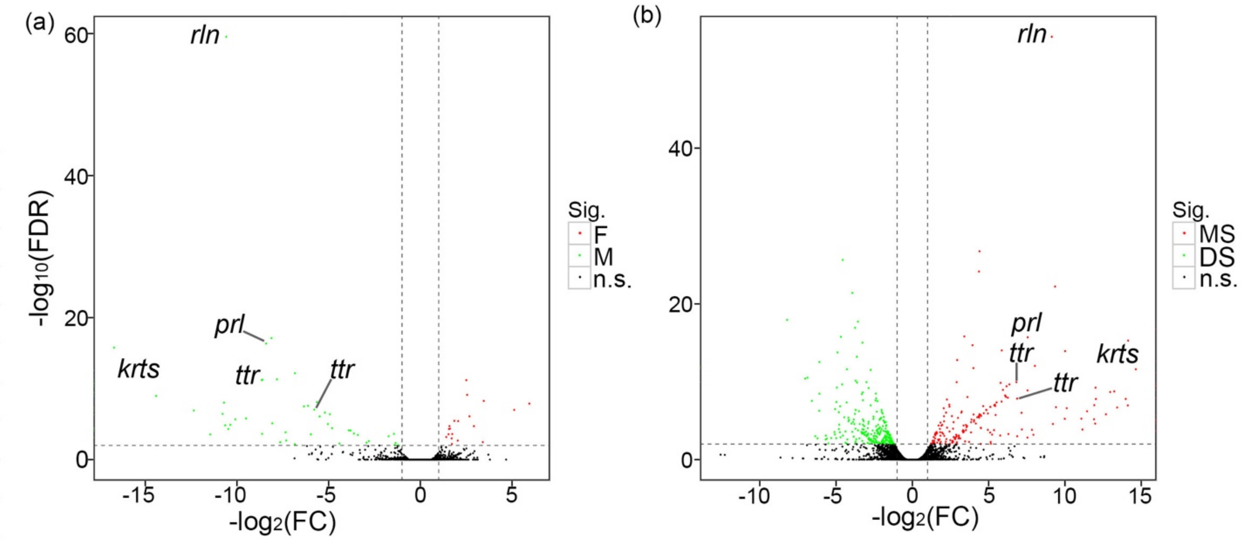

Supplementary Figure 12. DEGs between males' and females' MS at stage B (a) and males' MS vs DS at stage B (b). x axis is $-\log_2$(fold change), y axis is $-\log_{10}$(fasle discovery rate). F: females, M: males; ns: not significant; *krt*s: keratin genes; *prl*: prolactin; *rln*: relaxin; *ttr*: transthyretin.

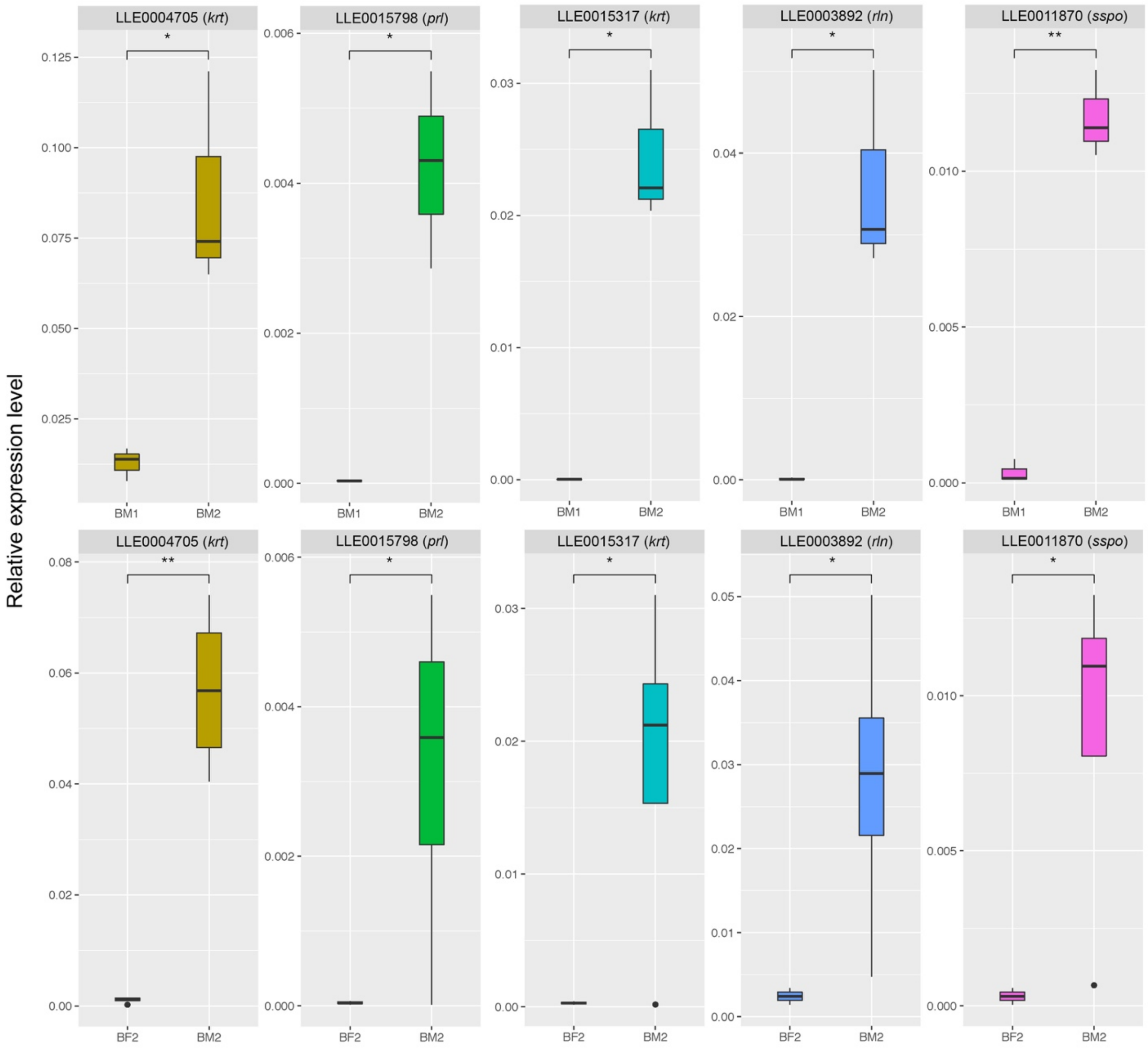

Supplementary Figure 13. Quantitative PCR validation for several DEGs. For the qPCR validation, we used MS and DS from adult males and adult females during the breeding season (BM1 vs BM2 and BM2 vs BF2). The expression levels of tested genes were normalized to the level of *gapdh* (glyceraldehyde-3-phosphate dehydrogenase). Two-sided Student's *t*-test was used to evaluate the significance of differential expression between samples. Primers are listed in Supplementary Table 12. Three biological replicates were examined for each sample. *sspo*: SCO-spondin (precursor).

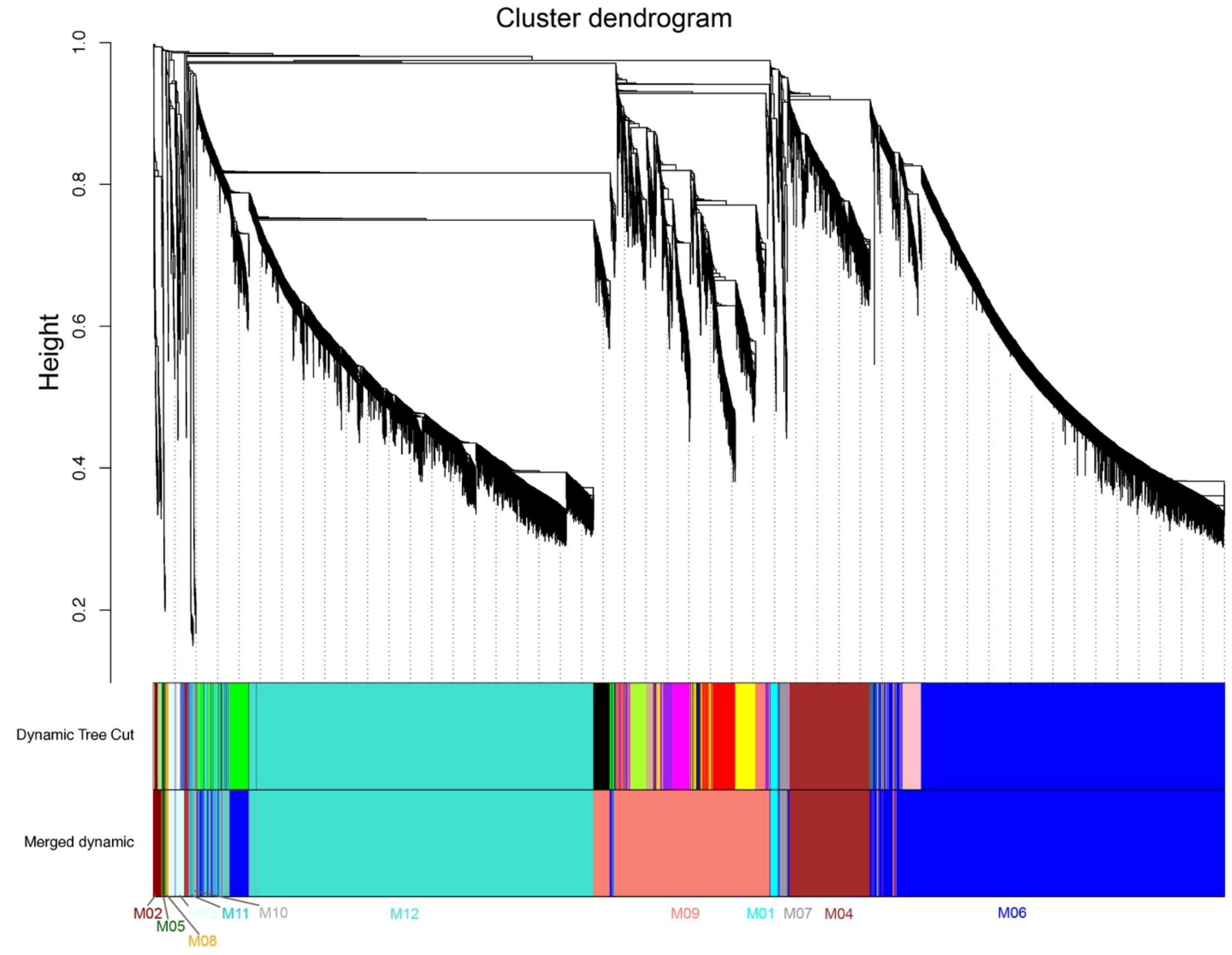

Supplementary Figure 14. Clustering dendrogram for module classification. Genes with similar coexpression patterns across samples were grouped using hierarchical clustering of dissimilarity among the topological overlap measures. Coexpressed modules were determined using a dynamic tree cutting algorithm setting with a minimum module size of 30 and a cut height of 0.998. Modules were assigned colors and names (ME01-12). ME13 includes genes that were not assigned to any module.

Supplementary Tables

Supplementary Table 1. Results from different assembling strategies.

Assembly methods	Contigs number	Contig length	Contig N50	contig Max	Scaffolds number	ScaffoldN50
Illumina (163×)+PacBio (10×) (IPA)	19,369	3,525,914,457	408,178	3,946,435	9,899	754,317
PacBio (80×)+Illumina (50×) polishing (PIP)	8,129	3,549,064,528	2,289,097	21,036,673	-	-
PacBio+Illumina polishing+Hi-C (PIH)	8,601	3,549,022,667	1,931,129	14,150,325	5,318	394,693,044

Supplementary Table 2. Summary of assembled pseudochromosomes.

Chr ID	Sequence Number	Sequence Length (bp)
Lle1	1,031	594,526,336
Lle2	793	489,609,153
Lle3	786	429,882,457
Lle4	797	422,459,683
Lle5	537	361,164,129
Lle6	816	214,688,685
Lle7	430	185,926,295
Lle8	264	173,733,504
Lle9	403	166,507,252
Lle10	237	137,667,082
Lle11	259	130,617,091
Lle12	225	117,428,518
Lle13	212	97,282,566

Supplementary Table 3. Genome assembly evaluated by Illumina reads and transcriptome alignment.

Illumina reads alignment	Selected samples	Total reads	Mapped reads	Properly mapped reads	Percentage
	R01	179,994,914	178,976,323	169,607,998	95.19%
	R02	181,882,576	180,811,093	170,949,026	94.95%
	R03	234,189,048	232,805,384	220,210,224	94.98%
	R04	213,847,796	212,441,888	201,100,922	94.99%
Transcriptome alignment*	Reads length (bp)	Total number	Aligned number	Percentage	
	All	258,442	257,391	99.59%	
	>=500	94,480	94,350	99.86%	
	>=1,000	44,135	44,080	99.88%	

Note: *: Range of read length: different ranges of read length; Total number: the number of transcripts within corresponding range; Aligned number: the number of transcripts aligned to the genome; Percentage: the percentage of aligned transcripts to total transcripts.

Supplementary Table 4. Statistics of the completeness of seven anurans based on 248 CEGs.

	Complete CEGs	detected complete CEGs from missing genes	Complete CEGs after adding missing genes	Percentage of complete CEGs out of 248 genes (%)
L. leishanense	208	33	241	97.18
N. parkeri	213	26	239	96.37
O. pumilio	176	19	195	78.63
R. catesbeiana	95	13	108	43.55
Rh. marina	204	9	213	85.89
*X. laevis**	241	-	-	97.18
X. tropicalis	229	8	237	95.56

Note: *we did not search missing genes in this allotetraploid species.

Supplementary Table 5. BUSCO evaluation across different anurans based on the same method.

Species	*L. leishanense*	*N. parkeri*	*O. pumilio*	*R. catesbeiana*	*Rh. marina*	*X. laevis**	*X. tropicalis*
Complete BUSCOs (C)	3,142 (79.54%)	3,325 (84.18%)	1,948 (49.32%)	1,142 (28.91%)	3,254 (82.38%)	3,627 (91.82%)	3,009 (76.18%)
Complete and single-copy BUSCOs (S)	3,080 (78.00%)	3,262 (82.58%)	1,921 (48.63%)	1,114 (28.20%)	3,178 (80.46%)	2,130 (53.92%)	2,974 (75.29%)
Complete and duplicated BUSCOs (D)	62 (1.60%)	63 (1.59%)	27 (0.68%)	28 (0.71%)	76 (1.92%)	1,497 (37.90%)	35 (0.89%)
Fragmented BUSCOs (F)	277 (7.01%)	245 (6.20%)	714 (18.08%)	830 (21.01%)	240 (6.08%)	105 (2.66%)	419 (10.61%)
Missing BUSCOs (M)	531 (13.44%)	380 (9.62%)	1,288 (32.61%)	1,978 (50.08%)	456 (11.54%)	218 (5.52%)	522 (13.22%)
Presence of missing BUSCOs	421	269	614	480	249	-	187
New complete and fragmented BUSCOs after adding presented Missing genes	3,840 (97.22%)	3,839 (97.19%)	3,276 (82.94%)	2,452 (62.08%)	3,743 (94.76%)	3,428 (86.78%)	3,615 (91.52%)
Total BUSCO groups searched	3,950	3,950	3,950	3,950	3,950	3,950	3,950

Note: *we did not search missing genes in this allotetraploid species.

Supplementary Table 6. The percentage of repetitive sequences in six anurans.

Type	Type	*X. tropicalis*	*N. parkeri*	*R. catesbeiana*	*L. leishanense*	*Rh. marina*	*O. pumilio*
RNA (retrotransposons)	LTR	4.01	6.08	13.89	17.53	16.19	14.37
	LINE	14.26	13.52	8.75	24.8	24.88	10.03
	PLE	7.62	4.34	9.37	6.88	3.15	3.74
	SINE	0.05	0.08	0.04	0.18	0.41	0.32
	TRIM	0.88	0.41	0.62	0.75	0.11	0.06
	LARD	14.91	25.04	15.79	3.48	9.37	13.89
DNA transposons	TIR	31.28	22.16	34.61	33.44	47.31	28.56
	Maverick	0.05	0.63	0.07	0.05	0.15	0.22
	Helitron	1.16	0.18	0.04	0.93	0.99	0.44
	Crypton	0.07	0.09	0.02	0.06	0.51	0.11
	MITE	0.03	0.11	0.05	0.29	0.96	0.28
Others	SSR	0.13	0.02	0.1	0.08	2.02	0.48
	Potential Host Gene	0.04	0.93	0.18	1.01	0.6	0.1
	Unknown	2.18	11.93	6.5	19.51	7.91	3.97
Total without overlap		51.88	56.71	66.9	77.05	86.58	62.94

Note: We constructed our own repeat sequence database employing LTR-FINDER[12], MITE-Hunter[13], RepeatScout 1.0.5[14] and PILER-DF[15]. The results were merged and classified using PASTEClassifier 1.0[16]. Then, the *de novo* constructed database was combined with the Repbase database 20.01 to create the final repeat library. Repeat sequences in *L. leishanense* genome were identified and classified using RepeatMasker 4.0.6[17]. The LTR family classification criterion was defined by which 5'LTR sequences of the same family would share at least 80% identity over at least 80% of their length. To compare repeat sequences across different anurans, we applied the same methods in other genomes (*N. parkeri*, *R. catesbeiana*, *X. tropicalis*, *Rh. marina* and *O. pumilio*). DIRS: Dictyostelium intermediate repeat sequence; LINE: long inter-spersed nuclear element; LTR: long terminal repeat; PLE: Penelope-like elements; SINE: short interspersed nuclear element; TIR: terminal inverted repeat.

Supplementary Table 7. The number of keratin genes in different species.

Species	Type I	Type II
L. leishanense	53	48
O. pumilio	35	20
R. catesbeiana	40	19
N. parkeri	49	25
Rh. marina	37	18
*X. laevis*_L	10	11
*X. laevis*_S	7	10
X. tropicalis	17	18
M. musculus	30	23
H. sapiens	28	26
A. carolinensis	20	25
D. rerio	13	3

Supplementary Table 8. DEG numbers for different pairwise comparisons.

Compared tissues	Pairwise comparisons	number of DEGs	All expressed genes
DS vs MS	AM1_AM2	64	14,789
	AF1_AF2	81	14,833
	BM1_BM2	447	14,468
	BF1_BF2	286	14,430
	CM1_CM2	854	14,596
	CF1_CF2	599	14,449
MS: males vs females	AM2_AF2	19	15,135
	BM2_BF2	83	14,409
	CM2_CF2	135	14,164
Brain: males vs females	AM3_AF3	37	15,794
	BM3_BF3	41	15,859
	CM3_CF3	39	15,712
A vs B	AM3_BM3	147	15,909
	AF3_BF3	15	15,796
B vs C	BM3_CM3	66	15,926
	BF3_CF3	52	15,693
Gonads: males vs females	AM4_AF4	8,415	16,807
	BM4_BF4	8,298	16,222
	CM4_CF4	10,093	16,551
A vs B	AM4_BM4	205	16,929
	AF4_BF4	109	14,186
B vs C	BM4_CM4	97	16,555
	BF4_CF4	256	14,315

Note: A, B, C mean developmental stages; M and F represent males and females; tissues 1-4 stands for DS, MS, brain and gonad.

Supplementary Table 9. Samples used for different sequencing methods.

Sequencing methods	Tissues	Gender and/or stages
Genomic PacBio sequencing	muscle	1 adult male
Illumina sequencing for genome size estimation	muscle	1 adult male
Transcriptomic sequencing for genome evaluation and protein-coding genes prediction	muscle, DS, MS, brain, testis, liver, heart, kidney, and spleen	1 adult male
HiC library preparation	whole blood cells	4 adult males
comparative transcriptomic sequencing	MS, DS, brain, gonads	Males and females at three developmental stages
qPCR validation	MS, DS	Males and females at stage B

Supplementary Table 10. Statistics of Illumina libraries.

Libraries	Data (bp)	Depth (X)	Q20(%)	Q30(%)
270 bp_1	26,727,028,500	7.64	95.04	89.37
270 bp_2	27,006,646,500	7.72	94.79	88.89
270 bp_3	34,775,527,800	9.94	94.84	89
270 bp_4	31,755,582,600	9.07	95.15	89.63
270 bp_5	26,948,378,400	7.70	95.6	90.56
270 bp_6	27,649,166,400	7.90	95.49	90.32
Sum of 270bp libraries	174,862,330,200	49.96	-	-
500 bp_1	34,174,438,768	9.76	91.79	85.1
500 bp_2	45,091,304,070	12.88	91.59	86.32
Sum of paired-end libraries	254,128,073,038	72.61	-	-
3 Kb_1	34,785,924,610	9.94	93.2	87.63
3 Kb_2	35,690,419,716	10.20	91.91	85.14
4 Kb_1	31,715,490,496	9.06	91.86	85.07
4 Kb_2	37,905,052,274	10.83	91.88	85.04
8 Kb_1	34,538,367,376	9.87	93.43	87.97
8 Kb_2	32,917,716,262	9.41	91.89	85.14
10 Kb_1	15,465,020,842	4.42	91.72	85.94
10 Kb_2	15,795,665,094	4.51	91.45	85.51
15 Kb_1	18,932,643,084	5.41	91.61	85.79
15 Kb_2	17,588,619,984	5.03	91.2	85.03
17 Kb_1	16,631,989,292	4.75	91.25	85.09
17 Kb_2	26,289,749,260	7.51	91.25	85.02
Sum of mate-pair libraries	318,256,658,290	90.93	-	-
Sum of Illumina libraries	572,384,731,328	163.54	-	-

Supplementary Table 11. Calibrated nodes used for dating the divergence time.

Nodes	Time (Ma)
(*X. laevis*_L; *X. laevis*_S) vs *N. parkeri*	187 - 220
(*X. laevis*_L; *X. laevis*_S) vs *D. rerio*	346-358
H. sapiens vs *M. musculus*	88-90
(*X. laevis*_L; *X. laevis*_S) vs *X. tropicalis*	34-79
*X. laevis*_L vs *X. laevis*_S	34 (internal control)

Note: the divergence time between *X. laevis* and *X. tropicalis* (34-79 Ma) was used as internal calibration node to evaluate the accuracy of estimation.

Supplementary Table 12. Primers used for quantitative PCR validation.

GeneID	Gene name	Forward primer (5'-3')	Revise primer (5'-3')	Target (bp)	Tm (℃)
LLE0004705	*krt*	ATGCTCGTGGTGGCGTTTC	TCTCCAGCCCAGCGTTTTC	162	62
LLE0015798	*prl*	GAAAGCGGTACAAATTGAGGAA	AAAAGGCAAACAGTCGAGCAT	164	62
LLE0015317	*krt*	GGGTTCGTTAGCAGAGGTCC	AAGTCGTTGATACGCTTGGTTC	136	62
LLE0003892	*rln*	TGTCATCGCACCCTATCACTG	GTATGGGCTAACCCTTTCCTG	157	60
LLE0011870	*sspo*	GTCAACTTCCACATCCAACACC	TATCCCGCCACGCACATA	174	60
LLE0022073	*gadph*	TAACGGTTTCGGTCGCATTG	TTTTCAGCCCTCACGGTCC	166	60/62

Supplementary References

1. Parra, G., Bradnam, K. & Korf, I. CEGMA: a pipeline to accurately annotate core genes in eukaryotic genomes. *Bioinformatics* **23**, 1061–1067 (2007).
2. Simão, F. A., Waterhouse, R. M., Ioannidis, P., Kriventseva, E. V. & Zdobnov, E. M. BUSCO: assessing genome assembly and annotation completeness with single-copy orthologs. *Bioinformatics* **31**, 3210–3212 (2015).
3. Burge, C. & Karlin, S. Prediction of complete gene structures in human genomic DNA. *J. Mol. Biol.* **268**, 78–94 (1997).
4. Stanke, M. & Waack, S. Gene prediction with a hidden Markov model and a new intron submodel. *Bioinformatics* **19 Suppl 2**, ii215-25 (2003).
5. Majoros, W. H., Pertea, M. & Salzberg, S. L. TigrScan and GlimmerHMM: two open source *ab initio* eukaryotic gene-finders. *Bioinformatics* **20**, 2878–2879 (2004).
6. Blanco, E., Parra, G. & Guigó, R. Using geneid to identify genes. *Curr. Protoc. Bioinforma.* **Chapter 4**, Unit 4.3 (2007).
7. Korf, I. Gene finding in novel genomes. *BMC Bioinformatics* **5**, 1–9 (2004).
8. Keilwagen, J. *et al.* Using intron position conservation for homology-based gene prediction. *Nucleic Acids Res.* **44**, (2016).
9. Kent, W. J. BLAT — The BLAST -Like Alignment Tool. *Genome Res.* **12**, 656–664 (2002).
10. Campbell, M. A., Haas, B. J., Hamilton, J. P., Mount, S. M. & Robin, C. R. Comprehensive analysis of alternative splicing in rice and comparative analyses with *Arabidopsis*. *BMC Genomics* **7**, 1–17 (2006).
11. Haas, B. J. *et al.* Automated eukaryotic gene structure annotation using EVidenceModeler and the program to assemble spliced alignments. *Genome Biol.* **9**, 1–22 (2008).
12. Xu, Z. & Wang, H. LTR-FINDER: an efficient tool for the prediction of full-length LTR retrotransposons. *Nucleic Acids Res.* **35**, 265–268 (2007).
13. Han, Y. & Wessler, S. R. MITE-Hunter: a program for discovering miniature inverted-repeat transposable elements from genomic sequences. *Nucleic Acids Res.* **38**, 1–8 (2010).
14. Price, A. L., Jones, N. C. & Pevzner, P. A. *De novo* identification of repeat families in large genomes. *Bioinformatics* **21**, 351–358 (2005).
15. Edgar, R. C. & Myers, E. W. PILER: identification and classification of genomic repeats. *Bioinformatics* **21**, 152–158 (2005).
16. Wicker, T. *et al.* A unified classification system for eukaryotic transposable elements. *Nat. Rev. Genet.* **8**, 973–82 (2007).
17. Tarailo-Graovac, M. & Chen, N. Using RepeatMasker to identify repetitive elements in genomic sequences. in *Current Protocols in Bioinformatics* **Chapter 4**, Unit 4.10 (2009).
