# Supplementary material for: Genomic and transcriptomic insights into molecular basis of sexually dimorphic nuptial spines in Leptobrachium leishanense
Source: Nat Commun. 2019 Dec 5;10:5551. doi: 10.1038/s41467-019-13531-5 (PMC6895153; doi:10.1038/s41467-019-13531-5)
Supplement: Supplementary file 2 — Description of Additional Supplementary Files [file 41467_2019_13531_MOESM2_ESM.pdf]

Description of Additional Supplementary Files

File Name: Supplementary Data 1
Description: Summary of chromosome rearrangements between *L. leishanense* and *X. tropicalis*.

File Name: Supplementary Data 2
Description: Significantly enriched biological processes and pathways for expanded gene families in seven anurans.

File Name: Supplementary Data 3
Description: Keratin genes identified in *L. leishanense* genome.

File Name: Supplementary Data 4
Description: Keratin gene expression values across different samples. The value [$\log_2$(TPM+0.1)] in each sample was the mean value of three duplicates.

File Name: Supplementary Data 5
Description: Gene IDs assigned in different co-expression modules.

File Name: Supplementary Data 6
Description: Significantly enriched biological processes and pathways for genes in different modules.

File Name: Supplementary Data 7
Description: Positively selected genes in *L. leishanense* compared with six other anurans.
