# Supplementary material for: Genomic and transcriptomic insights into molecular basis of sexually dimorphic nuptial spines in Leptobrachium leishanense
Source: Nat Commun. 2019 Dec 5;10:5551. doi: 10.1038/s41467-019-13531-5 (PMC6895153; doi:10.1038/s41467-019-13531-5)
Supplement: Supplementary file 10 — Reporting Summary [file 41467_2019_13531_MOESM10_ESM.pdf]

nature research

Corresponding author(s): Hua Wu

Last updated by author(s): Aug 27, 2019

Reporting Summary

Nature Research wishes to improve the reproducibility of the work that we publish. This form provides structure for consistency and transparency in reporting. For further information on Nature Research policies, see Authors & Referees and the Editorial Policy Checklist.

Statistics

For all statistical analyses, confirm that the following items are present in the figure legend, table legend, main text, or Methods section.

n/a	Confirmed	
☐	☒	The exact sample size (*n*) for each experimental group/condition, given as a discrete number and unit of measurement
☒	☐	A statement on whether measurements were taken from distinct samples or whether the same sample was measured repeatedly
☐	☒	The statistical test(s) used AND whether they are one- or two-sided<br>*Only common tests should be described solely by name; describe more complex techniques in the Methods section.*
☒	☐	A description of all covariates tested
☐	☒	A description of any assumptions or corrections, such as tests of normality and adjustment for multiple comparisons
☒	☐	A full description of the statistical parameters including central tendency (e.g. means) or other basic estimates (e.g. regression coefficient) AND variation (e.g. standard deviation) or associated estimates of uncertainty (e.g. confidence intervals)
☐	☒	For null hypothesis testing, the test statistic (e.g. *F*, *t*, *r*) with confidence intervals, effect sizes, degrees of freedom and *P* value noted<br>*Give P values as exact values whenever suitable.*
☒	☐	For Bayesian analysis, information on the choice of priors and Markov chain Monte Carlo settings
☐	☒	For hierarchical and complex designs, identification of the appropriate level for tests and full reporting of outcomes
☐	☒	Estimates of effect sizes (e.g. Cohen's *d*, Pearson's *r*), indicating how they were calculated

Our web collection on statistics for biologists contains articles on many of the points above.

Software and code

Policy information about availability of computer code

Data collection	Samples used for collecting different data had been listed in Supplementary Table 1. Softwares used for genome assembly: Canu v1.5, WTDBG v1.1.006; Pilon v1.22; BWA v0.7.1, LACHESIS. Softwares used for repetitive sequences prediction: LTR-FINDER, MITE-Hunter, RepeatScout v1.0.572 and PILER-DF,PASTEClassifier v1.0, RepeatMasker v4.0.6. Softwares used for protein-coding genes annotation: Genscan v1.0, Augustus v2.5.5, GlimmerHMM v3.0.1, GeneID v1.3, SNAP, GeMoMa v1.4.2, PASA v2.0.4, EVidenceModeler v1.1.1.
Data analysis	Chromosome synteny analysis: MCScanX, Circos v0.69.<br>Comparative genomic analyses: OrthoMCL v2.0.9, MUSCLE v3.8.31, RAxML v7.2.8, MCMCTree program in PAML v4.9, CAFÉ v3.1, CODEML in PAML v4.9.<br>Transcriptome analyses: DESeq package in R v3.5.1, WGCNA 1.63 in R v3.5.1, Cytoscape v3.6.1.

For manuscripts utilizing custom algorithms or software that are central to the research but not yet described in published literature, software must be made available to editors/reviewers. We strongly encourage code deposition in a community repository (e.g. GitHub). See the Nature Research guidelines for submitting code & software for further information.

Data

Policy information about availability of data

All manuscripts must include a data availability statement. This statement should provide the following information, where applicable:

- Accession codes, unique identifiers, or web links for publicly available datasets
- A list of figures that have associated raw data
- A description of any restrictions on data availability

All the data has been deposited in the NCBI database under the BioProject PRJNA505224. Specifically, the assembled version IPA of the the Leishan moustache toad genome has been deposited in NCBI Genbank (accession: RXON00000000). The version described in this paper is version RXON01000000. The HDF5 raw data for PacBio sequencing have been deposited in NCBI SRA database (accessions: SRR8897348-SRR8897543; SRR9670029-SRR9670067). The RNA-seq reads for 72 samples have been deposited in NCBI SRA database (accessions: SRR8736149-SRR8736220). The Hi-C library reads (including eight libraries) have been deposited in the SRA (accessions: SRR8784800-SRR8784807). The Illumina paired-end reads have been deposited in the SRA (accessions: SRR8788204-SRR8788209;

SRR10019514-SRR10019515). The Illumina mate-pair reads have been deposited in the SRA (accessions: SRR10019502-SRR10019513). The annotation files can be found in Figshare (https://figshare.com/; DOI: 10.6084/m9.figshare.8019986). Other miscellaneous information are available from the corresponding authors upon request. Published genome data used in the analyses can be found under the following accession codes and hyperlinks: X. tropicalis (GCF_000004195.3 [https://www.ncbi.nlm.nih.gov/assembly/GCF_000004195.3/]); X. laevis (GCF_001663975.1 [https://www.ncbi.nlm.nih.gov/assembly/GCF_001663975.1/]); N. parkeri (GCF_000935625.1 [https://www.ncbi.nlm.nih.gov/assembly/GCF_000935625.1/]); R. catesbeiana (GCA_002284835.2 [https://www.ncbi.nlm.nih.gov/assembly/GCA_002284835.2]); O. pumilio ([https://academic.oup.com/mbe/article/35/12/2913/5106668#supplementary-data]); Rh. marina (DOI:10.5524/100483 [http://gigadb.org/dataset/100483]); D. rerio (GCF_000002035.6 [https://www.ncbi.nlm.nih.gov/assembly/GCF_000002035.6/]); A. carolinensis (AnoCar2.0 [ftp://ftp.ensembl.org/pub/release-90/fasta/anolis_carolinensis/dna/]); M. musculus (GRCm38 [ftp://ftp.ensembl.org/pub/release-90/fasta/mus_musculus/dna/]); H. sapiens (GRCh38 [ftp://ftp.ensembl.org/pub/release-90/fasta/homo_sapiens/dna/]).

Field-specific reporting

Please select the one below that is the best fit for your research. If you are not sure, read the appropriate sections before making your selection.

☒ Life sciences ☐ Behavioural & social sciences ☐ Ecological, evolutionary & environmental sciences

For a reference copy of the document with all sections, see nature.com/documents/nr-reporting-summary-flat.pdf

Life sciences study design

All studies must disclose on these points even when the disclosure is negative.

Sample size	We did not perform sample size calculation. For transcriptome sequencing and analyzing, we used three duplications for each condition. Detailed sampling strategies have been listed in Supplementary Table 1.
Data exclusions	No data was excluded from the analyses.
Replication	No replication in this manuscript.
Randomization	No randomization in this manuscript.
Blinding	No blinding in this manuscript as the data were not allocated into groups.

Reporting for specific materials, systems and methods

We require information from authors about some types of materials, experimental systems and methods used in many studies. Here, indicate whether each material, system or method listed is relevant to your study. If you are not sure if a list item applies to your research, read the appropriate section before selecting a response.

Materials & experimental systems

n/a	Involved in the study	
☒	☐	Antibodies
☒	☐	Eukaryotic cell lines
☒	☐	Palaeontology
☐	☒	Animals and other organisms
☒	☐	Human research participants
☒	☐	Clinical data

Methods

n/a	Involved in the study	
☒	☐	ChIP-seq
☒	☐	Flow cytometry
☒	☐	MRI-based neuroimaging

Animals and other organisms

Policy information about studies involving animals; ARRIVE guidelines recommended for reporting animal research

Laboratory animals	The study did not involve laboratory animals.
Wild animals	For genome sequencing, we captured one adult male Leishan Moustache toad.<br>For transcriptome sequencing, we captured toads at three stages: sub-adult, adult at breeding season, and adult after breeding based on the morphological characteristics of nuptial spines and their occurring time. Both males and females were sampled at three stages.<br>The animals were caught near to their breeding stream. To collect tissues for genome and transcriptome sequencing, the captive toads were sacrificed by pithing.
Field-collected samples	This study did not involve laboratory work with field-collected samples.
Ethics oversight	All experiments on animals involved in this study have been approved by the Animal Ethics Committee in Central China Normal University (CCNU-IACUC-2019-008).

Note that full information on the approval of the study protocol must also be provided in the manuscript.
